# Glycemic control and weight loss benefits of fixed-ratio combinations of basal insulins and GLP1 receptor agonists in type 2 diabetes: A systematic review and network meta-analysis

**DOI:** 10.1007/s12020-026-04857-4

**Published:** 2026-10-31

**Authors:** A B M Kamrul-Hasan, Sunetra Mondal, Lakshmi Nagendra, Ibrahim Khalil, Deep Dutta, Joseph M Pappachan

**Affiliations:** 1 Department of Endocrinology, Mymensingh Medical College, Mymensingh, 2200 Bangladesh; 2 Department of Endocrinology, NRS Medical College, 138 AJC Bose Road, Kolkata, 700014 West Bengal India; 3 Department of Endocrinology, JSS Medical College, JSS Academy of Higher Education and Research, Mysuru, 570015 Karnataka India; 4 Department of Medicine, Ibrahim Khalil, MBBS, Dhaka Medical College and Hospital, Dhaka, 1000 Bangladesh; 5 Department of Endocrinology, CEDAR Superspeciality Healthcare, Dwarka, Delhi 110075 New Delhi, India; 6 Department of Endocrinology, Countess of Chester Hospital NHS Trust, CH2 2AZ Chester, UK; 7 Faculty of Science, Manchester Metropolitan University, Manchester, Greater Manchester, M15 6BH UK; 8 Department of Endocrinology, Kasturba Medical College, Manipal Academy of Higher Education, Manipal, Manipal, 576104 Karnataka India

**Keywords:** Basal insulin/GLP1 receptor agonist fixed-ratio combinations, IcoSema, IGlarLixi, IDegLira, HbA1c, Body weight

## Abstract

**Purpose:**

To compare glycemic and weight effects of fixed-ratio combinations (FRCs) of basal insulin (BI) and glucagon-like peptide-1 receptor agonists (GLP1RAs) in adults with type 2 diabetes (T2D).

**Methods:**

Randomized controlled trials of BI–GLP1RA FRCs in adults with T2D were identified across major databases through 20 December 2025. Co-primary outcomes were changes in glycated hemoglobin (HbA1c) and body weight. A frequentist random-effects network meta-analysis, with BI as the reference, was conducted in R using netmeta. Certainty was assessed using Confidence in Network Meta-Analysis (CINeMA).

**Results:**

Thirty trials involving 17,862 participants were included. All FRCs reduced HbA1c and body weight more than BI alone. For HbA1c, HR17031 had the largest estimated reduction (mean difference [MD]: -0.72%), followed by IDegLira (-0.64%) and iGlarLixi (-0.53%). For body weight, IcoSema had the largest estimated reduction (-3.16 kg), followed by HR17031 (-1.74 kg) and IDegLira (-1.65 kg). No HR17031 comparison with other FRCs was statistically significant for either co-primary outcome; its ranking is hypothesis-generating. Excluding the high-risk-of-bias trial produced broadly consistent estimates. Compared with BI, FRCs increased achievement of HbA1c < 7%, often without weight gain or clinically significant/severe hypoglycemia. CINeMA indicated predominantly low- to moderate-certainty evidence for FRC versus BI comparisons and low- to very low-certainty evidence for many indirect comparisons among active regimens.

**Conclusions:**

BI–GLP1RA FRCs improve glycemic control and weight outcomes compared with BI alone. Rankings and apparent differences among FRCs or versus other active regimens warrant cautious interpretation given heterogeneity, inconsistency, and limited certainty of indirect evidence.

**Graphical Abstract:**

**Figure:**
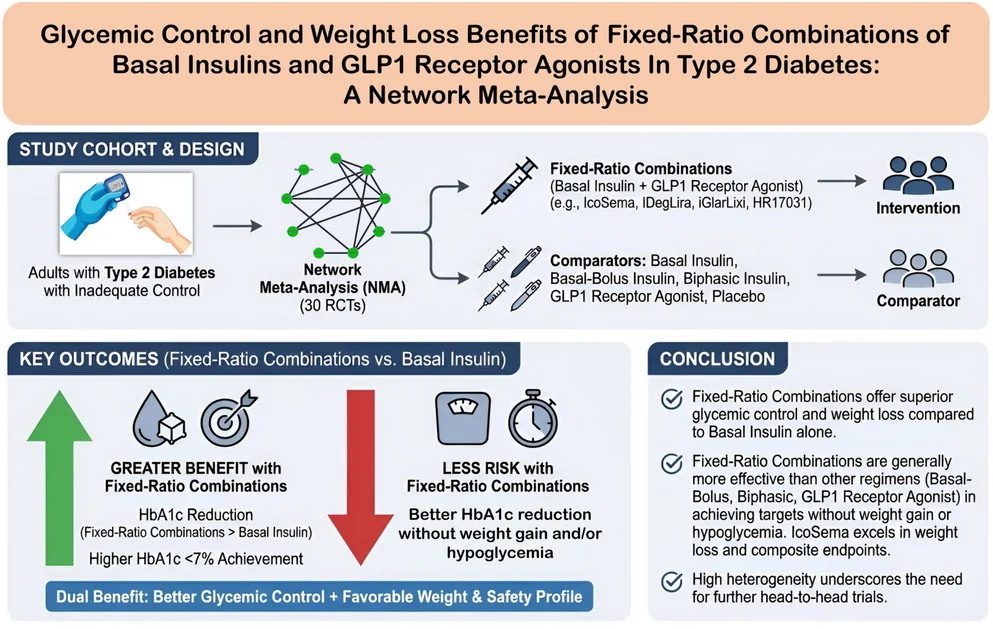

**Supplementary Information:**

The online version contains supplementary material available at https://doi.org/10.1007/s12020-026-04857-4.

## Introduction

Type 2 diabetes mellitus (T2D) is a growing global health challenge, impacting over 500 million people worldwide, with projections indicating it will continue to rise through 2050 [1]. Achieving optimal glycemic control while minimizing adverse effects, particularly weight gain and hypoglycemia, remains a central challenge in diabetes management. Combining basal insulin (BI) with glucagon-like peptide-1 receptor agonists (GLP1RAs) offers a complementary strategy that targets multiple pathophysiological mechanisms underlying T2D [2]. This approach addresses both the ongoing decline in beta-cell function and insulin resistance. BI consistently suppresses hepatic glucose production and helps manage fasting hyperglycemia, which is crucial for patients with declining beta-cell function. However, using BI alone has limited success in controlling post-meal glucose spikes and often leads to weight gain and a higher risk of hypoglycemia. GLP1RAs boost glucose-dependent insulin release, inhibit glucagon, slow gastric emptying, and curb appetite—mechanisms that help regulate blood sugar, support weight loss, and decrease hypoglycemia risk. Combining BI with GLP1RAs at a fixed ratio offers a logical intensification approach, likely providing better glycemic control than BI alone while avoiding the weight gain and hypoglycemia typical of basal-bolus (BB) insulin therapies [3–5].

Several randomized controlled trials (RCTs) show a significant HbA1c reduction of 1.5% to 2.5% with this combination, often surpassing BI or GLP1RA alone and matching BB insulin. BI and GLP1RA combinations offer fewer injections, lower hypoglycemia risk, and less glucose variability. This combination also leads to modest weight loss (up to 5 kg) compared with the weight gain seen with insulin monotherapy. However, heterogeneity across trial designs, including varying patient populations, baseline glycemic control, study durations, and insulin regimens, limits the interpretability of individual trials and complicates direct comparisons [6, 7]. Few meta-analyses have been conducted in this area, but traditional meta-analyses cannot thoroughly compare BI–GLP1RA combinations with different BI alone, premixed insulins, basal-plus, or BB regimens due to indirect comparisons and the absence of direct head-to-head data [8–12]. Additionally, existing meta-analyses have compared the effects of this molecule to BI, basal-plus, or BB regimens, while the potential benefits of this molecule compared to GLP1RAs have largely remained unexplored, although there are RCTs with GLP1RAs as comparators. Moreover, most existing meta-analyses were published before 2020, predating the emergence of newer fixed-ratio agents such as insulin icodec–semaglutide (IcoSema) and the publication of updated evidence on established combinations, including insulin degludec–liraglutide (IDegLira) and insulin glargine–lixisenatide (iGlarLixi), as well as contemporary insulin formulations.

Network meta-analysis (NMA) overcomes these challenges by combining direct and indirect evidence from all available trials. It allows comparisons between interventions, even when no direct head-to-head trials exist, and ranks interventions by effectiveness. This approach is particularly valuable when a common comparator links otherwise disparate trial networks [13]. This network meta-analysis will systematically synthesize RCTs evaluating fixed-ratio combinations (FRCs) of BI and GLP1RAs in adults with T2D, using BI (any formulation) as the primary reference treatment. By anchoring comparisons to BI, the analysis will quantify the incremental benefits of adding a GLP1RA, rank available FRCs for glycemic control and weight loss, and estimate the relative effects of other relevant regimens (BB and biphasic insulins, GLP1RA monotherapy, and placebo) versus BI. The overarching objective is to delineate the magnitude of benefit of FRCs over BI monotherapy and to inform contemporary treatment algorithms for T2D.

## Methods

This NMA was prospectively registered with PROSPERO (CRD420251242577), and the protocol summary is available online. It was conducted in accordance with the Cochrane Handbook for Systematic Reviews of Interventions [14], and its reporting adhered to the PRISMA-NMA guidelines [15]. No separate ethical approval was required for this NMA because approval had already been obtained for the individual RCTs included.

### Search strategy

Several databases and registers, including PubMed, Scopus, Web of Science, and ClinicalTrials.gov, were systematically searched, and Google Scholar was used to identify additional articles. The search covered these sources from inception through 20 December 2025. Using the Boolean operators “AND” and “OR,” the following terms were searched: “type 2 diabetes mellitus,” “type 2 diabetes,” “non-insulin-dependent diabetes,” “T2DM,” “T2D,” “basal insulin,” “long-acting insulin,” “background insulin,” “insulin degludec,” “insulin glargine,” “insulin detemir,” “insulin icodec,” “glucagon-like peptide 1 receptor agonist,” “glucagon-like peptide-1,” “GLP-1 receptor agonist,” “GLP-1 analogue,” “liraglutide,” “semaglutide,” “dulaglutide,” “exenatide,” “lixisenatide,” “albiglutide,” “efpeglenatide,” “taspoglutide,” “fixed-ratio combination,” “fixed dose combination,” “co-formulation,” “combination therapy,” “iGlarLixi,” “IDegLira,” “iDegLira,” “IcoSema,” “insulin glargine–lixisenatide,” “insulin degludec–liraglutide,” “insulin icodec–semaglutide,” “randomized controlled trial,” and “clinical trial.” The search targeted titles, abstracts, and keywords, with no language restrictions, to identify published studies. The full search strategies are included in Supplementary Table 1. Additionally, the process included reviewing references cited in the gathered articles and in relevant journals.

### Study selection

**Fig. 1:**
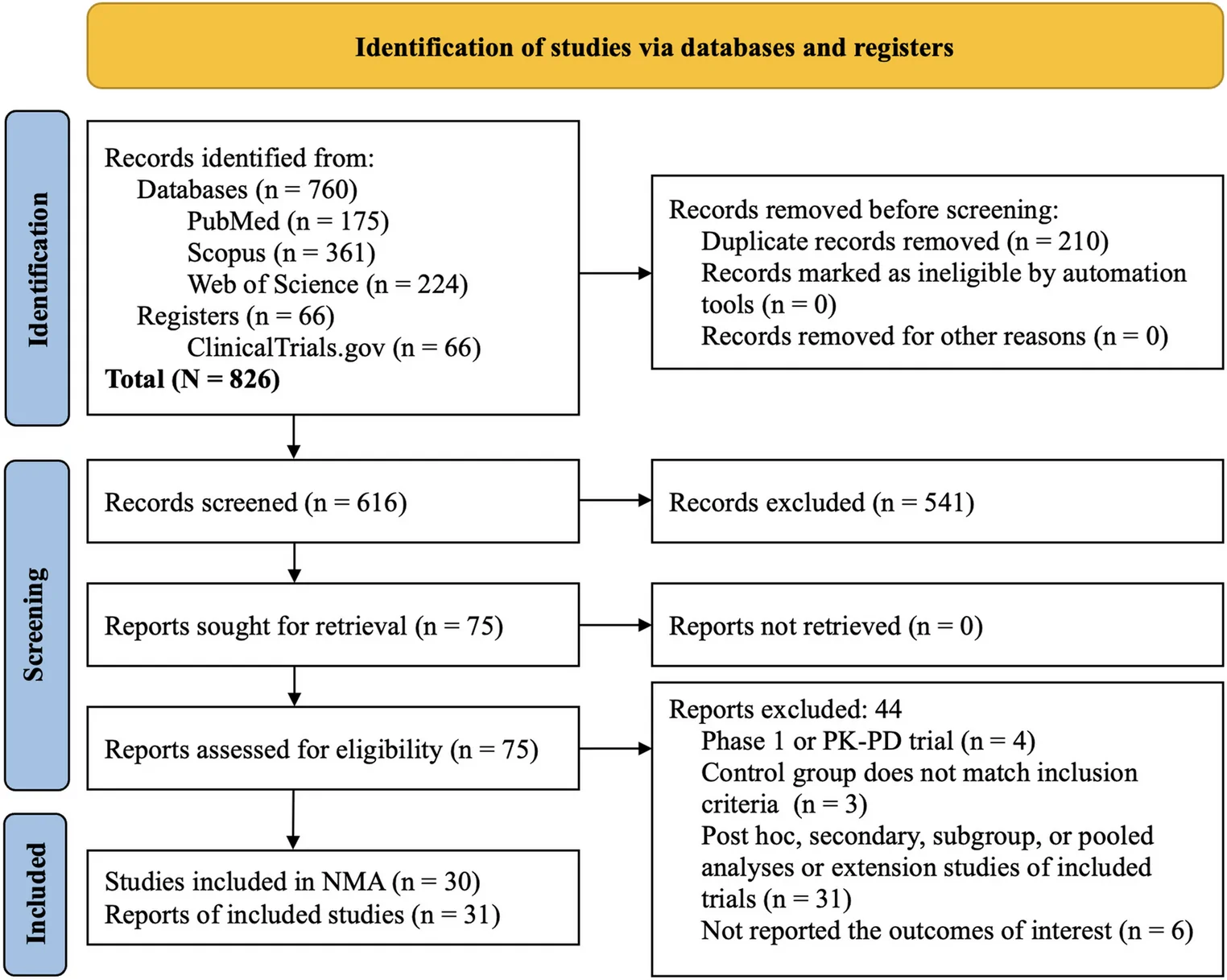
PRISMA 2020 flow diagram of study selection. Flow diagram showing identification, screening, eligibility assessment, and inclusion of randomized controlled trials in the network meta-analysis. Thirty randomized controlled trials, reported in 31 publications and involving 17,862 participants, were included

The Population, Intervention, Comparison, Outcomes, and Study (PICOS) framework was used to formulate eligibility criteria for the clinical trials in this NMA. The patient population (P) included adults with T2D who had inadequate glycemic control on existing glucose-lowering medications (GLMs). The intervention (I) group received any BI–GLP1RA FRC; the control (C) group received insulin (basal, biphasic, or BB), GLP1RA, or placebo for T2D management. Trials comparing the effects of two BI–GLP1RA FRCs were also considered. Outcomes (O) included changes in glycated hemoglobin (HbA1c) and body weight from baseline to the end of the study. We focused solely on RCTs (S) lasting at least 12 weeks and including participants aged 18 or older. RCTs must have at least two arms: one with any BI–GLP1RA FRC, with or without background therapy, and another with insulin (basal or biphasic or BB) or GLP1RA or a placebo, with or without background therapy. Excluded were nonrandomized trials, phase 1 RCTs, pharmacokinetic/pharmacodynamic studies, retrospective studies, post hoc, secondary, subgroup, or pooled analyses, or extension studies of included trials, conference proceedings, letters, case reports, articles lacking outcome data, animal or healthy human trials, and RCTs under 12 weeks. Three independent authors removed duplicates, screened titles and abstracts based on criteria, and then reviewed full texts to assess eligibility. The authors resolved disagreements by consensus and documented reasons for exclusion, as shown in the PRISMA flow diagram (Fig. 1).

### Outcomes analyzed

The co-primary outcomes were changes in glycated hemoglobin (HbA1c) and body weight from baseline to the end of the study. The secondary outcomes included the change in fasting plasma glucose (FPG) from baseline and the proportions of study participants who achieved HbA1c < 7%, as well as the composite outcomes of HbA1c < 7.0% without weight gain, HbA1c < 7.0% without clinically significant or severe hypoglycemic episodes, and HbA1c < 7.0% without weight gain and clinically significant or severe hypoglycemic episodes.

### Data extraction and dealing with missing data

Three authors independently extracted data using standard forms. Patient characteristics, including demographic details from both included studies, were recorded. For the review, we extracted data from all eligible studies on several key factors: name of the trial with the clinical trial unique identifier, first author, publication year, baseline GLMs, study arms, sample size, proportion of male participants, mean age, baseline HbA1c, body weight, duration of T2D, and study duration. Data on both primary and secondary outcomes were also extracted. For continuous outcomes, we extracted mean differences (MDs) and standard deviations (SDs) between baseline and end-of-study values. When SDs for change outcomes were not reported, they were derived from reported standard errors (SEs), confidence intervals (CIs), p‑values, or baseline and end‑of‑study SDs using standard formulas implemented in the web‑based Meta‑Analysis Accelerator [16]. For binary outcomes, we analyzed the available intention‑to‑treat or full‑analysis populations as reported in the original trials and did not impute missing event counts. No additional imputations were performed for missing continuous or binary outcome data beyond these standard transformations. We considered the specific BI–GLP1RA FRC separately; different basal, biphasic, and BB insulin regimens, as well as GLP1RAs used in the trials, were grouped accordingly. The supplementary materials were carefully reviewed, and all relevant information was obtained via email communication with the corresponding author (where necessary) and incorporated into the meta-analysis. Key numerical data, including screening and randomization numbers and analyses of the intention-to-treat, as-treated, and per-protocol populations, were meticulously examined. Attrition rates, including dropouts, follow-up losses, and withdrawals, were thoroughly investigated.

### Risk of bias assessment

Three authors independently assessed risk of bias (RoB) using version 2 of the Cochrane risk-of-bias tool for randomized trials (RoB 2) [17]. The RoB 2 domains cover all types of bias currently recognized as affecting RCT results, including bias arising from randomization, deviations from intended interventions, missing outcome data, outcome measurement, and selection of reported results. The RoB judgment categorized each domain as low RoB, some concerns, or high RoB. The least favorable assessment across the bias domains was used to determine the overall RoB for the result [17]. Disagreements were settled through consensus. Publication bias was assessed visually using comparison-adjusted funnel plots, followed by Egger’s test for a quantitative assessment [18].

### Data synthesis and statistical analysis

A frequentist random-effects network meta-analysis was performed in R version 4.4.2 (R Foundation for Statistical Computing, Vienna, Austria) using RStudio version 2025.09.2 + 418 (Posit Software, PBC, Boston, MA, USA), primarily with the netmeta package [19]. Treatment effects for both primary and secondary outcomes were estimated as MDs for continuous outcomes and odds ratios [ORs] for binary outcomes, along with 95% CIs, for each comparison against the reference treatment, i.e., BI (with placebo retained as a separate node where available). Network plots were produced to visualize the structure and connectivity of the treatment comparisons, illustrating direct and indirect evidence pathways. Pairwise comparisons for all treatment pairs were generated to provide comprehensive effect estimates from the NMA. Inconsistency analyses were conducted to evaluate the agreement between direct and indirect evidence across the network, utilizing the ‘netsplit’ function with the back-calculation method. Treatment effects were ranked from best to worst along the leading diagonal, and the results were presented as league tables. P-scores were also calculated to rank treatments by relative effectiveness, with higher P‐scores indicating greater effectiveness. Statistical significance was set at the 0.05 level. Statistical heterogeneity was evaluated using Cochran’s Q test and the I^2^ statistic [20]. The restricted maximum-likelihood estimator was used to estimate tau^2^, and I^2^ was calculated from Q. Values of I^2^ at 25%, 50%, and 75% indicate low, moderate, and high heterogeneity, respectively [21].

### Exploratory post-hoc analyses

Although not prespecified in the PROSPERO protocol, exploratory post-hoc analyses were conducted for the co-primary outcomes. Univariable random-effects network meta-regression was performed in R using the netmetareg() function of the netmeta package. Each potential effect modifier was evaluated in a separate model. For change in HbA1c, study-level baseline HbA1c and trial duration were assessed; for change in body weight, study-level baseline body weight and trial duration were assessed. Study-level covariates were calculated as sample-size-weighted means across treatment arms and were mean-centered before analysis. A common treatment-by-covariate interaction was assumed across comparisons. The models retained the treatment-node definitions, effect measures, basal-insulin reference group, random-effects framework, and REML estimator used in the primary NMA.

We also conducted a post hoc sensitivity analysis excluding trials judged to be at high overall risk of bias using RoB 2. All arms from excluded trials were removed before generating pairwise contrasts, and the NMA was repeated using the same analytical specifications as in the primary analysis. Robustness was assessed by comparing FRC-versus-basal-insulin effect estimates and 95% CIs, heterogeneity, inconsistency, and treatment rankings with those of the primary analysis. A further sensitivity analysis excluding trials with derived change-score SDs was considered but not undertaken: such SD derivation was required for 26 of 30 trials for both outcomes, and exclusion would have removed all IcoSema and HR17031 evidence and resulted in a sparse, non-representative network. Because these analyses were post hoc and the meta-regressions used aggregate study-level data, all findings were considered exploratory and hypothesis-generating.

### Assessment of the certainty of evidence

The certainty of evidence was systematically assessed using the Confidence In Network Meta-Analysis (CINeMA) framework [22]. This framework, which operationalizes confidence assessment for network meta-analysis estimates in line with GRADE principles, evaluates six domains: within-study bias, across-study bias, indirectness, imprecision, heterogeneity, and incoherence. Judgments across these domains informed an overall certainty rating for each comparison (high, moderate, low, or very low) [22].

## Results

### Search results

Figure 1 shows the study selection process. An initial search yielded 826 articles; after screening titles, abstracts, and full texts, this was reduced to 75. Ultimately, 30 RCTs with 31 published reports involving 17,862 participants, with follow-up durations ranging from 12 to 104 weeks, that met all predefined criteria were included in this NMA [23–53].

### Characteristics of included studies

Table 1 summarizes the included RCTs. Among the 30 included RCTs, Kawaguchi 2024 [40] did not report its trial phase; LixiLan PoC [44] and Ji 2025 [53] were phase 2 trials; VARIATION 2 SA [52] was a phase 4 trial; and the remaining 26 were phase 3 RCTs. Kawaguchi 2024 compared two BI-GLP1 FRCs, IDegLira with iGlarLixi [40]. The other 29 trials used BI-GLP1 FRCs in the intervention group; of those, three used IcoSema [23–25], 13 used IDegLira [26–39], 12 used iGlarLixi [41–52], and one used HR17031 [53]. Six studies included both BI and GLP1RA components of the respective BI–GLP1RA FRCs in the control arms [27–29, 48, 49, 53]; 13 used the BI component of the respective BI–GLP1RA FRCs [23, 30–32, 35, 37–39, 41, 43, 44, 46, 47, 52]; three used BB insulins [25, 26, 36]; two used biphasic insulins [50, 51]; four used GLP1RAs [24, 33, 42, 45]; and one trial used placebos [34] in the control group. Follow-up periods in the RCTs ranged from 12 to 104 weeks.

**Table 1:** Summary of the included trials and baseline characteristics of the trials’ participants

Study [Reference]	Trial reg. no., Trial phase	Authors, Publication year	Country	Baseline GLM	Treatment	*N*	HbA1c (%, mean)	Body weight (kg, mean)	BMI (kg/m^2^, mean)	Age (years, mean)	Male (%)	Duration of diabetes (years, mean)	Duration (weeks)
COMBINE 1 [23]	NCT05352815, Phase 3	Mathieu et al. 2025	20 countries/ regions	BI ± oral GLMs	IcoSema	646	8.22	83.7	29.7	60.5	62	15.2	52
					BI (icodec)	645	8.22	85.3	30.1	60.7	62	15.5	
COMBINE 2 [24]	NCT05259033, Phase 3	Lingvay et al. 2025	13 countries/ regions	GLP1RA s ± oral GLMs	IcoSema	342	8.07	87.58	30.58	59.9	58.8	12.94	52
					GLP1RA (semaglutide)	341	7.93	90.82	31.65	58.3	57.5	12.34	
COMBINE 3 [25]	NCT05013229, Phase 3	Billings et al. 2025	14 countries	BI ± oral GLMs	IcoSema	340	8.3	85.3	30.3	60.2	59	14.2	52
					BB insulin (glargine + aspart)	339	8.29	86.2	30.5	59.1	59	14.6	
DUAL HIGH [26]	NCT03737240, Phase 3	Galindo et al. 2023	USA	2–3 oral GLMs ± basal insulin	IDegLira	72	10.8	93.1	32.4	54.5	39	< 20: 86.2%; >20: 13.8%	26
					BB insulin (degludec + aspart)	73	10.7	91.9	31.5	53.8	48	< 20: 87.7%; >20: 12.3%	
DUAL I [27]	NCT01336023, Phase 3	Gough et al. 2014	19 countries	Metformin ± pioglitazone	IDegLira	833	8.3	87.2	31.2	55.1	52	6.6	26
					BI (degludec)	413	8.3	87.4	31.2	54.9	48	7	
					GLP1RA (liraglutide)	414	8.3	87.4	31.3	55	50	7.2	
DUAL I CHINA [28]	NCT03172494, Phase 3	Wang et al. 2022	China	Metformin ± one additional oral GLM	IDegLira	361	8.2	74.8	27.0	54.5	60.7	8	26
					BI (degludec)	179	8.31	73.2	26.5	55.7	55.9	8.63	
					GLP1RA (liraglutide)	180	8.21	73.8	26.5	54.1	60	8.05	
DUAL I JAPAN [29]	NCT02607306, Phase 3	Kaku et al. 2019	Japan	One oral GLM	IDegLira	275	8.5	70.7	26.1	56.9	70.5	8.1	52
					BI (degludec)	271	8.5	72.6	26.6	57.8	72	8.7	
					GLP1RA (liraglutide)	273	8.3	72.2	26.5	56.8	70.3	8.4	
DUAL II [30]	NCT01392573, Phase 3	Buse et al. 2014	7 countries	BI + metformin ± sulfonylurea or glinide	IDegLira	199	8.7	95.4	33.6	57	56	10	26
					BI (degludec)	199	8.8	93.5	33.8	58	53	11	
DUAL II CHINA [31]	NCT03175120, Phase 3	Pei et al. 2021	China	BI + metformin ± one additional oral GLM	IDegLira	302	8.93	76.8	27.5	54.5	60.6	11.52	26
					BI (degludec)	151	8.96	74.3	27.0	55.3	60.3	11.33	
DUAL II JAPAN [32]	NCT02911948, Phase 3	Watada et al. 2019	Japan	Basal or premixed insulin + metformin ± one additional oral GLM	IDegLira	105	8.61	73.9	27.3	56.6	66.7	14.33	26
					BI (degludec)	105	8.56	75.5	28.1	55.5	60	13.77	
DUAL III [33]	NCT01676116, Phase 3	Linjawi et al. 2017	5 countries	GLP1RA + metformin ± pioglitazone and/or sulfonylurea	IDegLira	292	7.8	95.6	32.9	58.3	52.4	10.4	26
					GLP1RA ^*^	146	7.7	95.5	33.0	58.4	48.6	10.4	
DUAL IV [34]	NCT01618162, Phase 3	Rodbard et al. 2017	7 countries	Sulfonylurea ± metformin	IDegLira	289	7.9	87.2	31.2	60	53.3	9	26
					Placebo	146	7.9	89.3	32.0	59.4	50.5	9.3	
DUAL V [35]	NCT01952145, Phase 3	Lingvay et al. 2016	10 countries	BI glargine + metformin	IDegLira	278	8.4	88.3	31.7	58.4	51.4	11.64	26
					BI (glargine)	279	8.2	87.3	31.7	59.1	49.1	11.33	
DUAL VII [36]	NCT02420262, Phase 3	Billings et al. 2018	12 countries	BI glargine + metformin ± additional oral GLMs	IDegLira	252	8.2	87.2	31.7	58.6	43.7	13.2	26
					BB insulin (glargine + aspart)	254	8.2	88.2	31.7	58	46.1	13.3	
DUAL VIII [37,38]	NCT02501161, Phase 3	Sesti et al. 2020, Aroda et al. 2019	16 countries	Oral GLMs	IDegLira	506	8.4	89.7	32.0	56.8	55	10	104^†^
					BI (glargine)	506	8.6	89	31.9	56.4	54	10.2	
DUAL IX [39]	NCT02773368, Phase 3	Philis-Tsimikas et al. 2019	11 countries	SGLT2 inhibitor ± oral GLMs	IDegLira	210	8.2	89.3	31.5	56.1	57.6	9.8	26
					BI (glargine)	210	8.4	87.2	30.9	57.2	60	9.3	
Kawaguchi 2024 [40]	UMIN000047518, Phase NR	Kawaguchi et al. 2024	Japan	Oral GLMs	iGlarLixi	18	8.5	67.4	26.2	63.2	55.6	10.4	18
					IDegLira	18	8.8	75.7	27.8	61.1	77.8	10.2	
LixiLan JP-L [41]	NCT02752412, Phase 3	Kaneto et al. 2020	Japan	BI and 1 or 2 GLMs	iGlarLixi	255	8.25	67.41	25.32	47.6	62.4	11.86	26
					BI (glargine)	257	8.28	65.93	24.88	48.1	57.2	12.02	
LixiLan JP-O1 [42]	NCT02749890, Phase 3	Watada et al. 2020	Japan	1 or 2 GLMs	iGlarLixi	161	8.39	72.26	26.79	58.3	64.6	8.12	26
					GLP1RA (lixisenatide)	160	8.38	72.99	26.85	57.7	63.7	9.22	
LixiLan JP-O2 [43]	NCT02752828, Phase 3	Terauchi et al. 2020	Japan	Up to 2 GLMs or up to 3 if a DPP-4 inhibitor was included	iGlarLixi	260	8.08	70.72	26.2	59.2	66.9	8.88	26
					BI (glargine)	260	8.01	69.1	25.87	60.2	64	9.61	
LixiLan PoC [44]	NCT01476475, Phase 2	Rosenstock et al. 2016	13 countries	Metformin monotherapy	iGlarLixi	161	8.1	90.1	32.2	56.9	51.3	6.3	24
					BI (glargine)	162	8	91.6	32.0	56.6	47.5	7.1	
LixiLan-G [45]	NCT02787551, Phase 3	Blonde et al. 2019	9 countries	GLP1RA + metformin ± pioglitazone/ SGLT2 inhibitor	iGlarLixi	252	7.9	93	32.8	59.2	49	11.2	26
					GLP1RA ^‡^	253	7.9	95.5	33.0	60	56	11	
LixiLan-L [46]	NCT02058160, Phase 3	Aroda et al. 2016	24 countries	BI ± oral GLMs	iGlarLixi	366	8.1	87.7	31.3	59.6	45	12	30
					BI (glargine)	365	8.1	87.1	31.0	60.3	48.5	12.1	
LixiLan-L-CN [47]	NCT03798080, Phase 3	Yuan et al. 2022	China	BI ± ≤ 2 oral GLMs	iGlarLixi	210	8.3	69.3	25.2	58.2	41.6	13.3	30
					BI (glargine)	211	8.3	69.8	25.3	56.7	43	11.4	
LixiLan-O [48]	NCT02058147, Phase 3	Rosenstock et al. 2016	23 countries	Metformin ± one additional oral GLM	iGlarLixi	468	8.2	89.4	31.6	58.2	52.7	8.9	30
					BI (glargine)	466	8.2	89.8	31.7	58.3	49.3	8.7	
					GLP1RA (lixisenatide)	233	8.3	90.8	32.0	58.7	43.2	8.9	
LixiLan-O-AP [49]	NCT03798054, Phase 3	Yang et al. 2022	4 countries/ territories	Metformin ± one additional oral GLM	iGlarLixi	348	8.4	70.6	26.1	55.4	51.3	8.03	24
					BI (glargine)	349	8.3	70.2	25.92	56.5	53.1	8.89	
					GLP1RA (lixisenatide)	175	8.3	71	26.12	56.3	54.2	8.93	
Soli-D [50]	NCT05413369, Phase 3	Liu et al. 2024	China	Metformin ± one additional oral GLM	iGlarLixi	291	8.58	70.72	25.92	56.3	51.5	8.56	24
					Biphasic (IDegAsp)	291	8.53	69.02	25.42	57.5	53.3	9.06	
SoliMix [51]	EudraCT 2017-003370-13, Phase 3	Rosenstock et al. 2021	17 countries	BI plus GLMs (metformin ± SGLT2 inhibitor)	iGlarLixi	443	8.6	80.7	29.7	59.8	50.6	13	26
					Biphasic (BIAsp)	444	8.6	82.2	30.0	59.8	49.1	13	
VARIATION 2 SA [52]	NCT03819790, Phase 4	Bajaj et al. 2022	Canada	Oral GLMs	iGlarLixi	51	8.3	76.2	28.5	60.5	52.9	13.9	12
					BI (glargine)	53	8.6	76.3	27.8	57.4	56.6	13.6	
Ji 2025 [53]	NCT05333835, Phase 2	Ji et al. 2025	China	Metformin ± one additional oral GLM	HR17031	183	8.5	70.2	25.6	55.9	59.6	6.8	26
					INS068	182	8.6	71.5	26.5	54.2	51.1	6.8	
					Noiiglutide	90	8.6	74.0	26.8	53.8	64.4	6.4	

^*^liraglutide/exenatide; ^†^HbA1c data at 26 weeks; ^‡^liraglutide/exenatide/dulaglutide/exenatide ER/albiglutide

BB, Basal-bolus; BI, Basal insulin; BMI, Body mass index; DPP-4, Dipeptidyl peptidase 4; FPG, Fasting plasma glucose; FRC, Fixed-ratio combinations; GLM, Glucose-lowering medications; GLP1RA, Glucagon-like peptide-1 receptor agonists; HbA1c, Glycated hemoglobin; IcoSema, Insulin icodec–semaglutide; IDegLira, Insulin degludec–liraglutide; iGlarLixi; insulin glargine–lixisenatide; NR, Not reported; MD, Mean difference; RCT, Randomized controlled trial; SGLT2, Sodium-glucose Cotransporter-2; T2D, Type 2 diabetes

Baseline characteristics, including mean HbA1c, BMI, diabetes duration, background glucose‑lowering therapy, and follow‑up duration, were broadly comparable across the main treatment arms, with overlapping ranges across the BI–GLP1RA FRCs, basal insulin, GLP1RA, basal‑bolus, and biphasic insulin arms. We did not formally assess transitivity quantitatively. Instead, we visually examined these distributions as a structured descriptive approach to key effect modifiers. As summarized in Table 1 for each trial arm, we used this to assess whether the transitivity assumption was reasonably plausible for the primary BI–GLP1RA FRC versus basal insulin comparisons. Control regimens were heterogeneous, ranging from BI alone to BB and biphasic insulin, GLP1RA monotherapy, and placebo. This heterogeneity may partly explain the high global heterogeneity and inconsistency and further limits the transitivity assumption for some indirect contrasts. Taken together, these overlapping distributions support a cautious application of the transitivity assumption for the primary BI–GLP1RA FRC versus basal insulin comparisons, while acknowledging that residual differences in trial populations and designs may still affect indirect comparisons. Supplementary Table 2 presents the outcomes for each study, including summary data for each intervention group.

### Risk of bias in the included studies

Supplementary Fig. 1 illustrates the specific and overall RoB in the 30 included RCTs. One trial (DUAL V) had a high overall RoB due to bias in the selection of the reported result [35]. 23 trials raised ‘some concerns’ about bias. The remaining six had low overall RoB. Although this trial may have contributed to overall heterogeneity and inconsistency, the direction of benefit for BI–GLP1RA FRCs versus basal insulin is consistent across multiple other trials, and this single high–RoB study does not drive our main conclusions. Assessment of publication bias using comparison-adjusted funnel plots for the changes in HbA1c (Supplementary Fig. 2A) and body weight (Supplementary Fig. 2B) revealed symmetric distributions of study effect sizes around zero for both outcomes. Egger’s regression tests were non-significant for HbA1c (P = 0.82) and body weight (P = 0.68), indicating no evidence of small-study effects or publication bias.

### Change in HbA1c

**Fig. 2:**
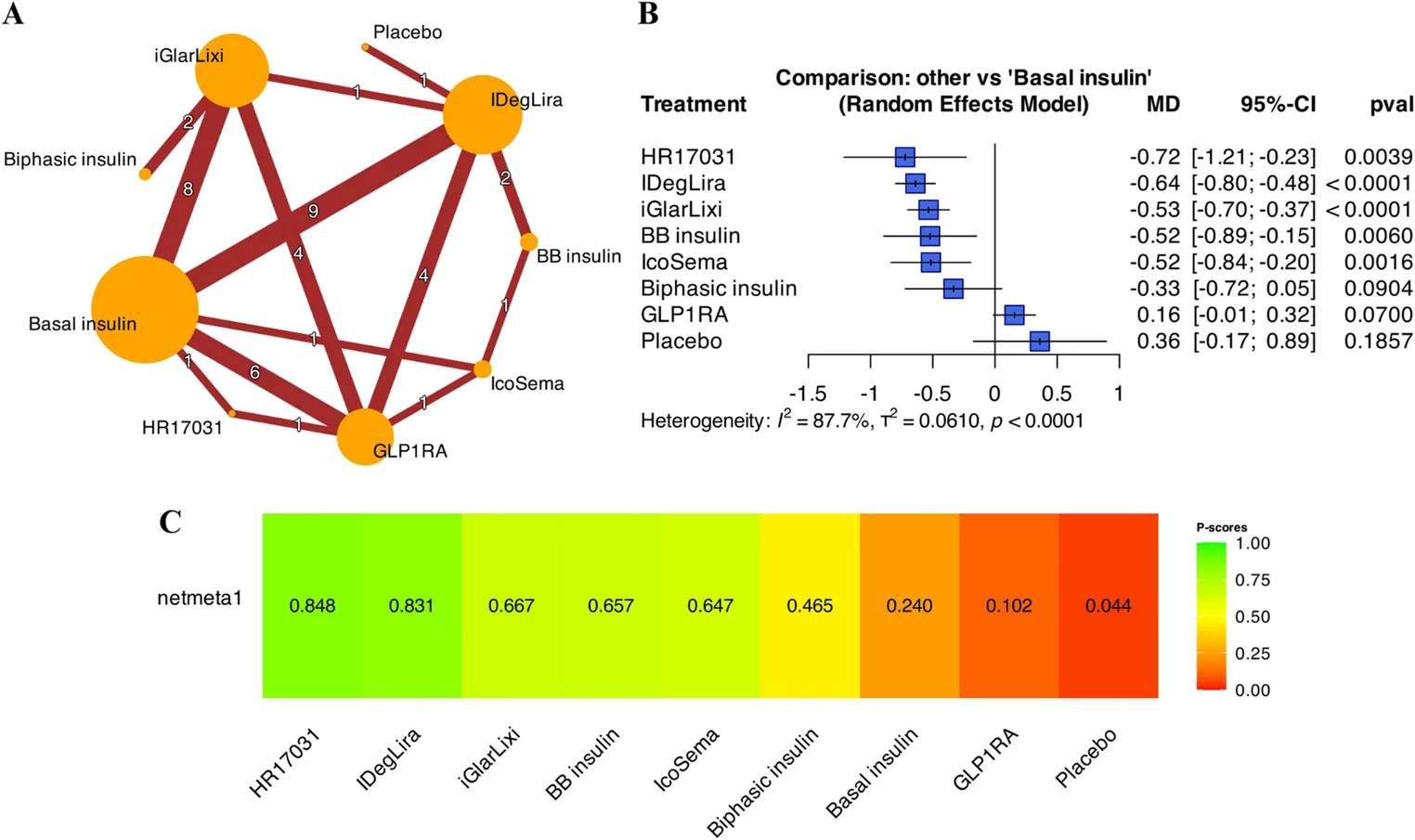
Network meta-analysis of change in glycated hemoglobin (HbA1c).** A** Network plot of the eligible treatment comparisons. Node size is proportional to the number of participants assigned to each treatment, and line thickness is proportional to the number of studies providing direct evidence; numbers on the lines indicate the number of studies. **B** Random-effects network meta-analysis forest plot showing mean differences (MDs) and 95% confidence intervals (CIs) for change in HbA1c relative to basal insulin. Negative MDs indicate a greater reduction in HbA1c than basal insulin. **C **P-scores for change in HbA1c. Higher P-scores indicate a higher relative ranking for HbA1c reduction; rankings should be interpreted cautiously because several indirect comparisons had low or very low certainty of evidence

The NMA included 30 studies, 9 treatments, 42 pairwise comparisons, and 17,862 observations, evaluating the impact of all treatments on changes in HbA1c relative to BIs. Figure 2A presents the network diagram, and Fig. 2B presents the NMA forest plot comparing all treatments with BIs; all BI–GLP1RA FRCs outperformed BIs. High heterogeneity was evident (τ² = 0.061, I² = 87.7%; total Q = 227.95, df = 28, *P* < 0.0001), driven by both within-design heterogeneity (Q = 139.35, df = 19, *P* < 0.0001) and between-design inconsistency (Q = 88.6, df = 9, *P* < 0.0001). Among the BI–GLP1RA FRCs, HR17031 (MD -0.72%, P score = 0.848) ranked top, followed by IDegLira (MD -0.64%, P score = 0.831) and iGlarLixi (MD -0.53%, P score = 0.667). All BI–GLP1RA FRCs ranked higher in HbA1c reduction than biphasic insulin and GLP1RA; HR17031, IDegLira, and iGlarLixi, but not IcoSema, were superior to BB insulin (Fig. 2B and C). Supplementary Table 3 provides the league table. In the netsplit analyses, most treatment contrasts showed broadly compatible direct and indirect estimates, with no statistically significant disagreement for the primary FRC versus basal insulin comparisons (Supplementary Fig. 3). However, the global inconsistency statistics indicate that although direct and indirect estimates were broadly compatible for the primary BI–GLP1RA FRC versus basal insulin contrasts, the degree of inconsistency suggests imperfect transitivity across the broader network. Consequently, more complex indirect comparisons and treatment rankings should be interpreted cautiously.

### Change in body weight

**Fig. 3:**
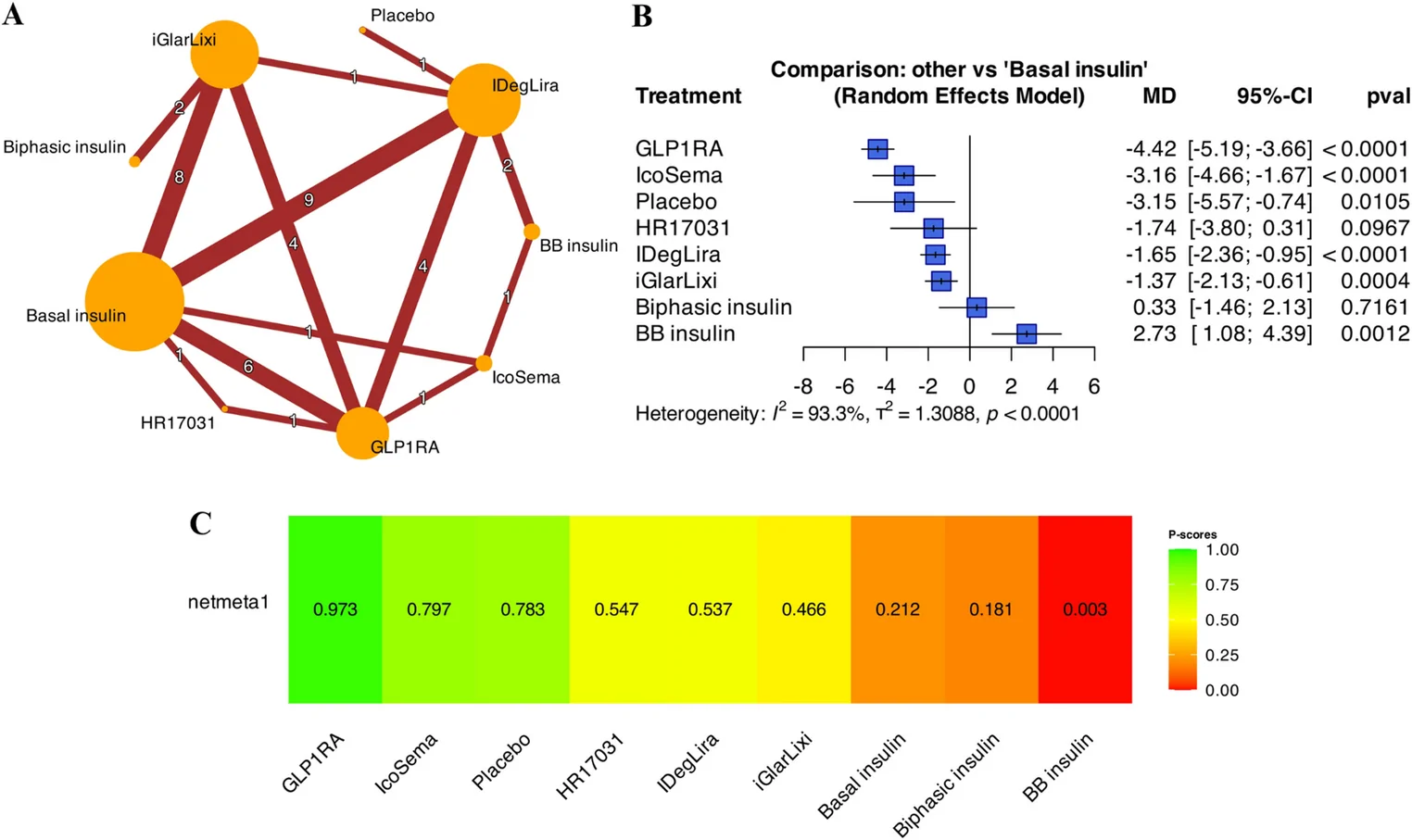
Network meta-analysis of change in body weight.** A** Network plot of the eligible treatment comparisons. Node size is proportional to the number of participants assigned to each treatment, and line thickness is proportional to the number of studies providing direct evidence; numbers on the lines indicate the number of studies. **B** Random-effects network meta-analysis forest plot showing mean differences (MDs) and 95% confidence intervals (CIs) for change in body weight relative to basal insulin. Negative MDs indicate greater weight loss, or less weight gain, than basal insulin. **C** P-scores for change in body weight. Higher P-scores indicate a higher relative ranking for weight reduction; rankings should be interpreted cautiously because of substantial heterogeneity, local inconsistency, and low-to-very-low certainty for many indirect comparisons

The NMA included 30 studies, 9 treatments, 42 pairwise comparisons, and 17,862 observations to evaluate the impact of all treatments on body weight changes relative to BIs. Figure 3A presents the network diagram, and Fig. 3B presents the NMA forest plot comparing all treatments with BIs; all BI–GLP1RA FRCs outperformed BIs. High heterogeneity was evident (τ² = 1.3088, I² = 93.3%; total Q = 414.6, df = 28, *P* < 0.0001), driven by both within-design heterogeneity (Q = 147.41, df = 19, *P* < 0.0001) and between-design inconsistency (Q = 267.56, df = 9, *P* < 0.0001). Among the BI–GLP1RA FRCs, IcoSema (MD -3.16 kg, P score = 0.797) had the top ranking in weight-lowering, followed by HR17031 (MD -1.74 kg, P score = 0.547) and IDegLira (MD -1.65 kg, P score = 0.537). BI–GLP1RA FRCs had a lower weight-loss potential than GLP1RAs, but higher than biphasic and BB insulin (Fig. 3B and C). Supplementary Table 4 provides the league table. For most treatment comparisons, direct and indirect estimates were in agreement (p for inconsistency ≥ 0.05); statistically significant disagreement between direct and indirect evidence was observed for IcoSema versus BI, iGlarLixi versus BI, GLP1RA versus IcoSema, and GLP1RA versus iGlarLixi, indicating local inconsistency in these contrasts (Supplementary Fig. 4). The very high inconsistency in body weight reflects tension between different segments of the network. This suggests that transitivity may not apply uniformly across all nodes and indicates that indirect comparisons between BI–GLP1RA FRCs and biphasic or basal‑bolus insulin regimens are less certain than the primary FRC versus basal insulin contrasts.

### Change in FPG

The NMA included 30 studies, 9 treatments, 42 pairwise comparisons, and 17,862 observations evaluating the impact of all treatments on changes in FPG relative to BIs. Supplementary Fig. 5A presents the network diagram, and Supplementary Fig. 5B presents the NMA forest plot comparing all treatments with BIs; only IDegLira outperformed BIs (MD -0.31 mmol/L, P score = 0.942). High heterogeneity was evident (τ² = 0.1134, I² = 77.3%; total Q = 123.53, df = 28, *P* < 0.0001), driven by both within-design heterogeneity (Q = 72.68, df = 19, *P* < 0.0001) and between-design inconsistency (Q = 50.85, df = 9, *P* < 0.0001). All BI–GLP1RA FRCs ranked higher than biphasic insulin and GLP1RA in FPG reduction; however, IDegLira, iGlarLixi, and HR17031, but not IcoSema, ranked higher than BB insulin (Supplementary Fig. 5B and 5 C). Supplementary Table 5 provides the league table. For most treatment comparisons, direct and indirect estimates were in agreement (p for inconsistency ≥ 0.05); statistically significant disagreement between direct and indirect evidence was observed only for GLP1RA versus iGlarLixi (Supplementary Fig. 5D).

#### Achieved HbA1c < 7%

The NMA included 28 studies with 9 treatments, 40 pairwise comparisons, and 17,722 observations, assessing the effect of all treatments compared to BIs on the proportion of subjects reaching HbA1c < 7%. Supplementary Fig. 6A presents the network diagram, and Supplementary Fig. 6B presents the NMA forest plot comparing all treatments with BIs; all BI–GLP1RA FRCs outperformed BIs. High heterogeneity was evident (τ² = 0.1801, I² = 82%; total Q = 144.45, df = 26, *P* < 0.0001), driven by both within-design heterogeneity (Q = 53.05, df = 18, *P* < 0.0001) and between-design inconsistency (Q = 91.4, df = 8, *P* < 0.0001). Among the BI–GLP1RA FRCs, iGlarLixi (OR 3.6, P score = 0.795) ranked top, followed by HR17031 (OR 3.7, P score = 0.777) and IcoSema (OR 3.5, P score = 0.776). All BI–GLP1RA FRCs ranked higher than BB insulin, biphasic insulin, and GLP1RA in achieving HbA1c < 7% (Supplementary Figs. 6B and 6 C). Supplementary Table 6 provides the league table. For most treatment comparisons, direct and indirect estimates were in agreement (p for inconsistency ≥ 0.05); statistically significant disagreement between direct and indirect evidence was observed only for iGlarLixi versus BI and GLP1RA versus iGlarLixi (Supplementary Fig. 6D).

#### HbA1c < 7% without weight gain

The NMA included 23 studies, 8 treatments, 35 pairwise comparisons, and 14,347 observations to evaluate the impact of all treatments relative to BIs on the proportion of study subjects who achieved HbA1c < 7% without weight gain. Supplementary Fig. 7A presents the network diagram, and Supplementary Fig. 7B presents the NMA forest plot comparing all treatments with BIs; iGlarLixi and IDegLira outperformed BIs. High heterogeneity was evident (τ² = 1.3347, I² = 96.9%; total Q = 715.58, df = 22, *P* < 0.0001), driven by both within-design heterogeneity (Q = 492.93, df = 17, *P* < 0.0001) and between-design inconsistency (Q = 222.65, df = 5, *P* < 0.0001). Among the BI–GLP1RA FRCs, HR17031 (OR 3.6, P score = 0.64) had the top ranking, followed by iGlarLixi (OR 3.0, P score = 0.602) and IDegLira (OR 2.8, P score = 0.59). Biphasic insulin ranked higher than any BI–GLP1RA FRCs. All BI–GLP1RA FRCs ranked higher than BB insulin; all but IcoSema were better than GLP1RA in achieving HbA1c < 7% without weight gain (Supplementary Fig. 7B and 7 C). Supplementary Table 7 provides the league table. The netsplit estimates showed no inconsistency between the indirect and direct effects (Supplementary Fig. 7D).

#### HbA1c < 7.0% without clinically significant or severe hypoglycemic episodes

The NMA included 23 studies with 8 treatments, 33 pairwise comparisons, and 15,222 observations, assessing how each treatment compares to BIs in achieving HbA1c < 7% without causing clinically significant or severe hypoglycemic episodes. Supplementary Fig. 8A presents the network diagram, and Supplementary Fig. 8B presents the NMA forest plot comparing all treatments with BIs; IcoSema, IDegLira, and iGlarLixi outperformed BIs. High heterogeneity was evident (τ² = 0.1741, I² = 83.7%; total Q = 128.76, df = 21, *P* < 0.0001), driven by both within-design heterogeneity (Q = 5.91, df = 14, *P* < 0.0001) and between-design inconsistency (Q = 76.85, df = 7, *P* < 0.0001). Among the BI–GLP1RA FRCs, IcoSema (OR 4.1, P score = 0.93) ranked top, followed by IDegLira (OR 3.4, P score = 0.852) and iGlarLixi (OR 2.7, P score = 0.700). All BI–GLP1RA FRCs ranked higher than biphasic insulin, GLP1RA, and BB insulin in achieving HbA1c < 7% without clinically significant or severe hypoglycemic episodes (Supplementary Figs. 8B and 8 C). Supplementary Table 8 provides the league table. For most treatment comparisons, direct and indirect estimates were in agreement (p for inconsistency ≥ 0.05); statistically significant disagreement between direct and indirect evidence was observed only for iGlarLixi versus BI and GLP1RA versus iGlarLixi (Supplementary Fig. 8D).

#### HbA1c < 7.0% without weight gain and clinically significant or severe hypoglycemic episodes

The NMA included 24 studies, 8 treatments, 36 pairwise comparisons, and 16,015 observations, evaluating the impact of all treatments relative to BIs on the proportion of study subjects who achieved HbA1c < 7% without weight gain and clinically significant or severe hypoglycemic episodes. Supplementary Fig. 9A presents the network diagram, and Supplementary Fig. 9B presents the NMA forest plot comparing all treatments with BIs; IcoSema, IDegLira, and iGlarLixi outperformed BIs. High heterogeneity was evident (τ² = 0.4284, I² = 89.4%; total Q = 217.76, df = 23, *P* < 0.0001), driven by both within-design heterogeneity (Q = 70.02, df = 17, *P* < 0.0001) and between-design inconsistency (Q = 147.74, df = 6, *P* < 0.0001). Among the BI–GLP1RA FRCs, IcoSema (OR 6.1, P score = 0.877) had the top ranking, followed by IDegLira (OR 3.9, P score = 0.647) and HR17031 (OR 3.4, P score = 0.599). Biphasic insulin ranked higher than any BI–GLP1RA FRCs. All BI–GLP1RA FRCs ranked higher than biphasic and BB insulin; IcoSema were better than GLP1RA in achieving HbA1c < 7% without weight gain and clinically significant or severe hypoglycemic episodes (Supplementary Figs. 9B and Supplementary 9 C). Supplementary Table 9 provides the league table. For most treatment comparisons, direct and indirect estimates were in agreement (p for inconsistency ≥ 0.05); statistically significant disagreement between direct and indirect evidence was observed only for iGlarLixi versus BI, GLP1RA versus IDegLira, and GLP1RA versus iGlarLixi (Supplementary Fig. 9D).

### Exploratory network meta-regression

In univariable random-effects network meta-regression, higher study-level baseline HbA1c was associated with relative treatment effects on change in HbA1c (𝛽 per 1%-point higher baseline HbA1c, + 0.8376; 95% CI, 0.2910 to 1.3843; *P* = 0.0027), with an estimated 28% reduction in between-study variance (τ^2^ = 0.0434). Trial duration was not associated with relative HbA1c effects (𝛽 per 1-week longer, + 0.0091; 95% CI, -0.0050 to + 0.0232; *P* = 0.2076), explaining 3% of the between-study variance.

For body weight, neither baseline body weight (𝛽 per 1 kg higher baseline weight, + 0.0558; 95% CI, -0.0014 to + 0.1130; *P* = 0.0559) nor trial duration (𝛽 per 1 week longer, + 0.0114; 95% CI, -0.0189 to + 0.0417; *P* = 0.4622) showed clear evidence of an association with relative treatment effects; the corresponding reductions in between-study variance were 10% and 0%, respectively. These post hoc analyses were based on aggregate study-level covariates and were considered exploratory (Supplementary Table 10).

### Post-hoc sensitivity analysis

After excluding DUAL V, which was judged to be at high overall risk of bias, 29 studies remained in the networks for both co-primary outcomes. For change in HbA1c, the estimated effects of the fixed-ratio combinations versus basal insulin remained favorable and were broadly consistent with those in the primary analysis: HR17031, MD -0.72% (95% CI -1.22 to -0.22); IDegLira, -0.64% (-0.81 to -0.47); iGlarLixi, -0.53% (-0.70 to -0.36); and IcoSema, -0.51% (-0.84 to -0.19) (Supplementary Fig. 12). Heterogeneity remained high (I^2^ = 88.1%, τ^2^ = 0.0638; *P* < 0.0001).

For changes in body weight, the estimated effects of the fixed-ratio combinations versus basal insulin were also directionally consistent with the primary analysis: IcoSema, MD -3.11 kg (95% CI -4.58 to -1.64); HR17031, -1.72 kg (-3.74 to 0.30); IDegLira, -1.50 kg (-2.23 to -0.76); and iGlarLixi, -1.34 kg (-2.10 to -0.59) (Supplementary Fig. 13). Heterogeneity remained high (I^2^ = 93.0%, τ^2^ = 1.2620; *P* < 0.0001). The relative ranking of the fixed-ratio combinations was broadly similar to that in the primary analysis.

### Certainty of evidence: confidence in network meta-analysis (CINeMA) assessment

CINeMA assessment of change in HbA1c showed overall low-to-moderate confidence across most comparisons (Supplementary Fig. 10). Within-study bias was the most frequent limitation, with several comparisons judged as having some concerns, leading to downgrading. Reporting bias and indirectness were generally not problematic. However, imprecision substantially affected comparisons supported by single or small studies, while heterogeneity contributed to downgrading in several multi-study contrasts. Incoherence was not a major concern. Direct comparisons between GLP1RA and IcoSema were rated with high confidence, whereas many indirect comparisons were rated low to very low confidence, mainly because of imprecision and heterogeneity. CINeMA assessment of change in body weight also indicated predominantly low-to-moderate confidence across direct comparisons (Supplementary Fig. 11). Within-study bias mainly drove downgrading, with most comparisons judged to have some concerns. Imprecision affected several contrasts supported by single studies, while heterogeneity further reduced confidence in multi-study comparisons, particularly those involving basal or biphasic insulin. Incoherence was more pronounced for this outcome, leading to additional downgrading across several direct and indirect estimates. Reporting bias and indirectness were generally not serious concerns. Overall, indirect comparisons were largely rated low to very low confidence. These certainty ratings indicate that the primary comparisons of BI–GLP1RA FRCs versus basal insulin are supported more strongly than indirect comparisons among individual FRCs, for which confidence was often low to very low. Accordingly, rankings and indirect contrasts for body weight, particularly those involving single-study nodes, should be interpreted cautiously.

## Discussion

This network meta‑analysis of 30 randomized controlled trials, including 17,862 adults with T2D, shows that BI–GLP1RA FRCs provide superior glycemic control and weight benefits compared with BI alone. CINeMA indicates predominantly low-to-moderate confidence for these outcomes and lower confidence for many indirect comparisons between active regimens. All evaluated FRCs lowered HbA1c more than BI, and most performed at least as well as BB insulin for glycemia while avoiding the typical insulin‑related trade-offs of weight gain and increased hypoglycemia. For body weight, FRCs produced weight loss relative to BI and more favorable profiles than biphasic and BB insulin, although GLP1RA monotherapy remained the most potent weight‑lowering option.

These findings extend earlier pairwise meta‑analyses by simultaneously ranking all BI–GLP1RA FRCs against BI, GLP1RA monotherapy, biphasic insulin, BB insulin, and placebo [8–12]. Previous work established that adding a GLP1RA to BI improves HbA1c and weight versus BI alone, but could not comprehensively compare individual FRCs or position them within a broader network of injectable therapies. By anchoring the analysis in BI and incorporating trials of long‑established FRCs (IDegLira, iGlarLixi) alongside newer investigational co‑formulations (IcoSema and HR17031), this study offers an integrated and contemporary assessment of the relative benefits and limitations of this therapeutic class.

The place of FRCs must be interpreted within the context of rapidly evolving treatment algorithms. Recent guidelines from major professional societies prioritize potent GLP1RAs and dual or triple incretin agonists, particularly for individuals with obesity and high cardiovascular risk, and consequently assign a more selective role to BI‑based intensification [54, 55]. In this landscape, BI–GLP1RA FRCs are unlikely to be first-line injectable therapy for most patients with T2D. However, moderate-certainty evidence supports their role as a useful insulin-based intensification option for selected patients for whom incretin-first strategies are contraindicated, poorly tolerated, inaccessible, or unaffordable, and for many older adults who may not require or tolerate maximally dosed incretin therapy [54, 55]. Our findings thus complement, rather than oppose, incretin-focused guidelines by guiding insulin‑based intensification when it is clinically necessary or structurally unavoidable. Global availability and regulatory status are key constraints. IDegLira and iGlarLixi are approved and marketed in various regions, while IcoSema and HR17031 remain investigational, have been tested only in a few clinical trials, and are not yet approved for routine clinical use. This limits the immediate practical application of their ranking for day‑to‑day prescribing, even though their inclusion improves the completeness of the evidence base and anticipates future therapeutic options. Furthermore, IDegLira and iGlarLixi are not widely accessible outside certain regions, and manufacturers are increasingly prioritizing obesity-targeted incretin therapies and free-combination treatments over fixed-dose BI–GLP1RA co-formulations. Nevertheless, the underlying concept of combining basal insulin and GLP1RA in a single injection remains relevant, particularly in low‑ and middle‑income settings where access to the latest dual- or triple-incretin agents is limited, but basal insulin and at least one GLP1RA are available. In such settings, FRCs, where marketed or structured BI plus GLP1RA regimens may offer a pragmatic alternative to BB or premixed insulin.

The specific value of FRCs lies in patients for whom treatment simplicity is paramount, particularly older adults [6, 7]. Progression from oral agents or BI‑augmented oral therapy to full BB insulin typically increases the number of injections, monitoring burden, hypoglycemia risk, and weight gain. These issues are especially problematic in frail elderly individuals, those with cognitive impairments, and patients with limited self-management ability. Our findings show that several FRCs can achieve guideline‑recommended HbA1c targets with weight loss or weight neutrality and without additional hypoglycemia compared with BI, while matching or exceeding the glycaemic efficacy of BB regimens. For such patients, a once‑daily FRC and, if ultimately approved and marketed, a once‑weekly co‑formulation such as IcoSema offer a “one‑shot” intensification strategy that can delay or avoid the need for complex multi‑injection insulin regimens.

When interpreting the rankings, it is important to consider both the heterogeneity of the included trials and the certainty of the underlying evidence. In particular, many indirect comparisons among individual FRCs were rated as having low to very low confidence by CINeMA and should therefore be regarded as exploratory rather than definitive. This uncertainty also applies to indirect comparisons between FRCs and other active regimens, including basal–bolus and biphasic insulin, where the certainty of evidence was low or very low. Exploratory network meta-regression suggested that baseline HbA1c may contribute to heterogeneity in HbA1c effects, whereas trial duration and the evaluated moderators for body-weight effects showed no clear association with relative treatment effects. Exclusion of DUAL V, the only trial judged at high overall risk of bias, yielded broadly similar FRC-versus-basal-insulin estimates for both co-primary outcomes. The included trials varied in baseline HbA1c, diabetes duration, concomitant glucose‑lowering medications, titration algorithms, and follow‑up durations (12–104 weeks). IDegLira and iGlarLixi are supported by extensive phase 3 programs across diverse populations and clinical scenarios, providing a large “information size” and more stable estimates. By contrast, HR17031 has been evaluated in a single 26‑week phase 2 trial in a geographically restricted population [53], and IcoSema, although studied in phase 3 programs [23–25], remains investigational without long‑term post‑marketing experience. As a result, the apparent HbA1c advantage of HR17031 over other FRCs in this network should be considered hypothesis‑generating rather than conclusive, because it is based on point estimates and P-scores from a single small phase 2 trial. Importantly, none of the pairwise network comparisons between HR17031 and the other FRCs reached statistical significance for HbA1c or body weight, despite numerically favorable estimates. Confidence in its ranking is lower than for IDegLira and iGlarLixi, and these results should not be applied in routine practice until larger, long-term phase 3 data and regulatory approval are available. A further distinction lies in the long‑term evidence base for individual components. Insulin degludec, insulin glargine, liraglutide, lixisenatide, semaglutide, and once‑weekly insulin icodec have all undergone extensive clinical development, including large cardiovascular and renal outcome trials for several GLP1RAs. Clinicians may reasonably place greater weight on FRCs built from components with established long‑term safety, cardiovascular benefit, and well‑defined pharmacokinetic profiles. In contrast, investigational co‑formulations without such mature data should be used only within clinical trials until their risk‑benefit balance is fully characterized and regulatory approval is granted.

This NMA has several limitations. Our search did not include Embase due to resource constraints. This represents a deviation from standard PRISMA/MECIR recommendations for comprehensive evidence identification and may have reduced the completeness of trial retrieval. Although we searched PubMed, Scopus, Web of Science, ClinicalTrials.gov, Google Scholar, and reference lists, and included 30 RCTs, the possibility that eligible studies were missed cannot be ruled out. The validity of indirect comparisons rests on the transitivity assumption—that key effect modifiers such as baseline HbA1c, BMI, diabetes duration, background glucose‑lowering therapy, type of control regimen, and follow‑up duration are reasonably similar across treatment contrasts. Although these characteristics broadly overlapped across arms in Table 1, using study-level data means that residual imbalances and effect modification by factors such as age, renal function, or cardiovascular status cannot be excluded. Comparator heterogeneity (basal insulin alone, basal‑bolus and biphasic insulin, GLP1RA monotherapy, and placebo) and the pooling of different basal insulin formulations (glargine, degludec, detemir, once‑weekly icodec) and diverse GLP1RAs (short‑ and long‑acting agents with varying potency) were necessary to maintain network connectivity but yield class‑level rather than drug‑specific estimates and pose a specific threat to strict transitivity, particularly for indirect comparisons between BI–GLP1RA FRCs and more complex insulin regimens. The very high global heterogeneity and inconsistency for HbA1c and body weight further reduce confidence in indirect estimates and strengthen the case for interpreting treatment rankings and between‑FRC contrasts as hypothesis‑generating rather than definitive, even though the direction of effect for the primary BI–GLP1RA FRC versus basal insulin comparisons is more consistent. A further limitation is that change-score SDs for most trials were derived from reported SEs or CIs. A sensitivity analysis excluding studies with derived change-score SDs could not be meaningfully conducted for the co-primary outcomes, as it would have removed most of the available evidence and produced a sparse, non-representative network. These limitations may contribute to residual uncertainty and help explain the imprecision and inconsistency reflected in the CINeMA assessments. Trial durations were predominantly short‑ to medium‑term, so the durability of dual benefits in HbA1c and weight, and their translation into hard outcomes such as cardiovascular events, kidney disease progression, and mortality, remain uncertain. Finally, economic outcomes, quality of life, and treatment satisfaction were not assessed, limiting inferences about the broader value and acceptability of BI–GLP1RA FRCs in routine care.

Despite these caveats, this work has notable strengths: a comprehensive, up‑to‑date evidence base; inclusion of all developed and investigational BI–GLP1RA FRCs; a prespecified protocol; established network meta-analytic methods; and a structured assessment of evidence certainty. Collectively, the results support BI–GLP1RA FRCs as an important option for insulin‑based intensification in adults with T2D, particularly when weight gain, hypoglycemia, and regimen complexity need to be minimized, and when access to or tolerability of newer incretin‑only or dual/triple-agonist therapies is limited.

## Conclusions

FRCs of BI and GLP1RAs provide better glycemic control and more favorable weight and hypoglycemia profiles than BI alone, with low- to moderate-certainty evidence supporting these conclusions for the main comparisons. Among these, IDegLira and iGlarLixi are supported by extensive phase 3 evidence and have already been approved in multiple regions, whereas IcoSema and HR17031 remain investigational and should be regarded as experimental until larger, longer-term data and regulatory approvals are available. Any apparent ranking advantage of HR17031 should be interpreted as hypothesis-generating rather than as established superiority. BI–GLP1RA FRCs should therefore be considered an important insulin-based intensification option for selected patients, particularly when weight gain, hypoglycemia, and regimen complexity need to be minimized, while recognizing that rankings among individual co-formulations are based largely on low-certainty indirect evidence.

## Supplementary information

Below is the link to the electronic supplementary material.

Supplementary Material 1

Supplementary Material 2

Supplementary Material 3

## Data Availability

No datasets were generated or analysed during the current study.

## Abbreviations

BB: Basal-bolus
BI: Basal insulin
CI: Confidence interval
CINeMA: Confidence In Network Meta-Analysis
DPP-4: Dipeptidyl peptidase 4
FPG: Fasting plasma glucose
FRC: Fixed-ratio combinations
GLM: Glucose-lowering medications
GLP1RA: Glucagon-like peptide-1 receptor agonists
HbA1c: Glycated hemoglobin
IcoSema: Insulin icodec–semaglutide
IDegLira: Insulin degludec–liraglutide
iGlarLixi: insulin glargine–lixisenatide
NMA: Network meta-analysis
OR: Odds ratio
PICOS: The Population, Intervention, Comparison, Outcomes, and Study design
PRISMA: Preferred Reporting Items for Systematic Reviews and Meta-Analyses
MD: Mean difference
RCT: Randomized controlled trial
RoB: Risk of bias
SD: Standard deviation
SGLT2: Sodium-glucose Cotransporter-2
T2D: Type 2 diabetes

## Declarations

### Registration information

The meta-analysis was registered in PROSPERO under the registration number CRD420251242577. The review protocol summary is available on the PROSPERO website (https://www.crd.york.ac.uk/PROSPERO/view/CRD420251242577).
