# Supplementary material for: Glycemic control and weight loss benefits of fixed-ratio combinations of basal insulins and GLP1 receptor agonists in type 2 diabetes: A systematic review and network meta-analysis
Source: Endocrine. 2026 Oct 31;91(1):366. doi: 10.1007/s12020-026-04857-4 (PMC13723511; doi:10.1007/s12020-026-04857-4)
Supplement: Supplementary file 1 [file 12020_2026_4857_MOESM1_ESM.pdf]

Supplementary Table 1. Search strings

**Database**	**Search strings**
**PubMed**	(("Diabetes Mellitus, Type 2"[Mesh] OR "type 2 diabetes mellitus"[tiab] OR "type 2 diabetes"[tiab] OR "non-insulin-dependent diabetes"[tiab] OR T2DM[tiab] OR T2D[tiab]))<br>AND<br>(("Insulin, Long-Acting"[Mesh] OR "basal insulin"[tiab] OR "long-acting insulin"[tiab] OR "background insulin"[tiab] OR "insulin degludec"[tiab] OR "insulin glargine"[tiab] OR "insulin detemir"[tiab] OR "insulin icodec"[tiab]))<br>AND<br>(("Glucagon-Like Peptide 1 Receptor Agonists"[Mesh] OR "glucagon-like peptide 1 receptor agonist"[tiab] OR "glucagon-like peptide-1"[tiab] OR "GLP-1 receptor agonist"[tiab] OR "GLP-1 analogue"[tiab] OR liraglutide[tiab] OR semaglutide[tiab] OR dulaglutide[tiab] OR exenatide[tiab] OR lixisenatide[tiab] OR albiglutide[tiab] OR efpeglenatide[tiab] OR taspoglutide[tiab]))<br>AND<br>(("Drug Combinations"[Mesh] OR "fixed-ratio combination"[tiab] OR "fixed dose combination"[tiab] OR "co-formulation"[tiab] OR "combination therapy"[tiab] OR iGlarLixi[tiab] OR IDegLira[tiab] OR iDegLira[tiab] OR IcoSema[tiab] OR "insulin glargine–lixisenatide"[tiab] OR "insulin degludec–liraglutide"[tiab] OR "insulin icodec–semaglutide"[tiab]))<br>AND<br>(("Randomized Controlled Trial"[Publication Type] OR "Controlled Clinical Trial"[Publication Type] OR "randomized controlled trial"[tiab] OR "clinical trial"[tiab] OR randomi*[tiab]))
**Scopus**	TITLE-ABS-KEY("type 2 diabetes mellitus" OR "type 2 diabetes" OR "non-insulin-dependent diabetes" OR T2DM OR T2D)<br>AND<br>TITLE-ABS-KEY("basal insulin" OR "long-acting insulin" OR "background insulin" OR "insulin degludec" OR "insulin glargine" OR "insulin detemir" OR "insulin icodec")<br>AND

	TITLE-ABS-KEY("glucagon-like peptide 1 receptor agonist" OR "glucagon-like peptide-1" OR "GLP-1 receptor agonist" OR "GLP-1 analogue" OR liraglutide OR semaglutide OR dulaglutide OR exenatide OR lixisenatide OR albiglutide OR efpeglenatide OR taspoglutide)<br>AND<br>TITLE-ABS-KEY("fixed-ratio combination" OR "fixed dose combination" OR "co-formulation" OR "combination therapy" OR iGlarLixi OR IDegLira OR iDegLira OR IcoSema OR "insulin glargine-lixisenatide" OR "insulin degludec-liraglutide" OR "insulin icodec-semaglutide")<br>AND<br>TITLE-ABS-KEY("randomized controlled trial" OR "clinical trial" OR randomi*)
**Web of Science**	TS=("type 2 diabetes mellitus" OR "type 2 diabetes" OR "non-insulin-dependent diabetes" OR T2DM OR T2D)<br>AND<br>TS=("basal insulin" OR "long-acting insulin" OR "background insulin" OR "insulin degludec" OR "insulin glargine" OR "insulin detemir" OR "insulin icodec")<br>AND<br>TS=("glucagon-like peptide 1 receptor agonist" OR "glucagon-like peptide-1" OR "GLP-1 receptor agonist" OR "GLP-1 analogue" OR liraglutide OR semaglutide OR dulaglutide OR exenatide OR lixisenatide OR albiglutide OR efpeglenatide OR taspoglutide)<br>AND<br>TS=("fixed-ratio combination" OR "fixed dose combination" OR "co-formulation" OR "combination therapy" OR iGlarLixi OR IDegLira OR iDegLira OR IcoSema OR "insulin glargine–lixisenatide" OR "insulin degludec–liraglutide" OR "insulin icodec–semaglutide")<br>AND<br>TS=("randomized controlled trial" OR "clinical trial" OR randomi*)

Supplementary Table 2. Study-level outcome data used in the network meta-analysis. Values for continuous outcomes are mean change from baseline and standard deviation (SD), unless otherwise stated. For dichotomous outcomes, N denotes the number of participants analyzed, and n denotes the number achieving the specified outcome. A negative value for HbA1c, body weight, or fasting plasma glucose indicates a reduction from baseline. Dashes indicate data not reported or not available for analysis.

Study	Treatment	N	HbA1c (%)		Body weight (Kg)		FPG (mmol/L)		HbA1c <7%		HbA1c <7.0% without clinically significant or severe hypoglycemic episodes		HbA1c <7.0% without weight gain		HbA1c <7.0% without weight gain and clinically significant or severe hypoglycemic episodes	
			Mean	SD	Mean	SD	Mean	SD	N	n	N	n	N	n	N	n
COMBINE 1	IcoSema	646	-1.55	0.76	-3.7	5.08	-1.68	2.29	646	463	646	428	-	-	646	360
	Basal insulin	645	-0.89	0.76	1.89	5.08	-1.54	2.29	645	229	645	196	-	-	645	66
COMBINE 2	IcoSema	342	-1.35	0.74	0.84	4.36	-2.48	2.03	342	251	342	244	-	-	342	103
	GLP1RA	341	-0.9	0.74	-3.7	4.6	-1.41	2.03	341	164	341	156	-	-	341	138
COMBINE 3	IcoSema	340	-1.47	0.92	-3.56	5.35	-1.56	2.4	340	227	340	214	340	16	340	170
	BB insulin	339	-1.4	1.1	3.16	5.89	-1.58	2.4	339	190	339	118	339	4	339	20
DUAL HIGH	IDegLira	72	-3.18	2.29	1.24	8.33	-5.1	7.2	72	33	72	19	72	13	72	8
	BB insulin	73	-3	1.79	5.84	6.18	-5.3	6.6	73	26	73	6	73	3	73	1
DUAL I	IDegLira	833	-1.9	1.1	-0.5	3.5	-3.6	3	833	671	833	503	833	385	833	296
	Basal insulin	413	-1.4	1	1.6	4	-3.6	3.56	413	269	413	169	413	87	413	58
	GLP1RA	414	-1.3	1.1	-3	3.5	-1.8	3.6	414	250	414	239	414	225	414	215
DUAL I CHINA	IDegLira	361	-1.66	0.88	0.1	2.7	-3.51	2.27	361	278	361	265	361	147	361	136
	Basal insulin	179	-1.13	0.99	1.2	2.7	-3.24	2.34	179	83	179	80	179	34	179	32
DUAL I CHINA	GLP1RA	180	-1.04	0.89	-2.4	2.7	-1.6	2.11	180	87	180	84	180	74	180	72

	IDegLira	275	-2.42	1.04	2.9	3.2	-4.08	2.47	275	245	270	227	275	44	270	37
DUAL I JAPAN	Basal insulin	271	-1.8	1.02	4.1	4.3	-3.97	2.56	271	189	265	169	271	22	265	22
	GLP1RA	273	-1.8	0.92	-1	3.4	-2.62	1.95	273	208	271	206	273	142	271	141
DUAL II	IDegLira	199	-1.9	1.09	-2.7	3.7	-3.5	2.9	199	119	199	97	199	101	199	80
	Basal insulin	199	-0.89	1.18	0	3.4	-2.6	3.3	199	46	199	31	199	24	199	17
DUAL II CHINA	IDegLira	302	-1.93	1.14	-0.7	3	-3.44	3	302	151	302	148	302	98	302	94
	Basal insulin	151	-1.06	1.19	0.4	2.7	-2.81	3.15	151	23	151	22	151	7	151	7
DUAL II JAPAN	IDegLira	105	-1.95	1.01	-0.7	3.5	-2.81	3.17	105	75	105	70	105	50	105	49
	Basal insulin	105	-0.65	0.98	0.7	2.7	-2.29	2.68	105	23	105	20	105	9	105	7
DUAL III	IDegLira	292	-1.3	0.8	2	3.9	-2.98	2.28	292	219	-	-	-	-	-	-
	GLP1RA	146	-0.3	0.9	-0.8	3	-0.6	2.74	146	53	-	-	-	-	-	-
DUAL IV	IDegLira	289	-1.5	0.8	0.5	3.1	-2.6	2.61	289	229	-	-	-	-	-	-
	Placebo	146	-0.5	0.8	-1	2.6	-0.31	2.43	146	42	-	-	-	-	-	-
DUAL V	IDegLira	278	-1.81	1.08	-1.4	3.5	-2.83	2.05	278	199	278	151	278	139	278	108
	Basal insulin	279	-1.13	0.98	1.8	3.6	-2.77	2.05	279	131	279	82	279	55	279	34
DUAL VII	IDegLira	252	-1.48	0.79	-0.93	0.22	-2.4	3.2	252	166	252	145	252	109	252	96
	BB insulin	254	-1.46	0.8	2.64	0.22	-1.9	3.68	254	170	254	85	254	39	254	16
DUAL VIII	IDegLira	506	-1.99	1.14	1.7	6.75	-3.93	2.96	506	281	506	262	506	400	506	101
	Basal insulin	506	-1.69	1.1	3.4	6.75	-3.73	2.75	506	144	506	129	506	474	506	31
DUAL IX	IDegLira	210	-1.94	0.95	0	3.8	-3.59	2.91	210	178	210	166	197	91	210	88
	Basal insulin	210	-1.68	1.05	2	3.9	-3.39	2.52	210	150	210	118	202	38	210	35
Kawaguchi 2024	iGlarLixi	18	-1.5	1.1	-2.5	2.7	-2.49	3.45	-	-	-	-	-	-	-	-
	IDegLira	18	-1.6	1.3	-1.8	3.4	-5.01	4.97	-	-	-	-	-	-	-	-
LixiLan JP-L	iGlarLixi	255	-1.27	0.94	-0.51	2.46	-0.81	2.25	255	132	255	103	255	89	255	69
	Basal insulin	257	-0.53	0.93	0.55	2.45	-0.77	2.26	257	41	257	35	257	24	257	21
Lixilan JP-O1	iGlarLixi	161	-1.58	0.91	0.62	2.25	-2.38	1.81	161	105	-	-	161	48	-	-
	GLP1RA	160	-0.51	0.92	-1.32	2.27	-0.09	1.85	160	31	-	-	160	28	-	-

Lixilan JP-O2	iGlarLixi	260	-1.4	0.68	-0.26	2.53	-1.77	1.52	260	186	260	156	260	92	260	80
	Basal insulin	260	-0.76	0.69	-1.33	2.53	-1.37	1.52	260	100	260	90	260	30	260	26
LixiLan PoC	iGlarLixi	161	-1.82	0.74	-0.97	3.67	-3.35	1.65	161	135	161	109	160	90	161	74
	Basal insulin	162	-1.64	0.73	0.48	3.59	-3.51	1.63	162	126	162	96	161	60	162	47
LixiLan-G	iGlarLixi	252	-1	0.79	1.89	3.49	-2.28	1.76	252	156	252	143	-	-	-	-
	GLP1RA	253	-0.4	0.8	-1.14	3.5	-0.61	1.77	253	66	253	64	-	-	-	-
LixiLan-L	iGlarLixi	366	-1.1	1.15	-0.7	3.83	-0.4	1.91	366	201	366	116	366	125	366	73
	Basal insulin	365	-0.6	1.15	0.7	3.82	-0.5	1.91	365	108	365	68	365	49	365	33
LixiLan-L-CN	iGlarLixi	210	-1.4	1.45	-0.3	2.9	-0.5	1.45	210	133	-	-	210	85	212	74
	Basal insulin	211	-0.7	1.45	0.7	2.91	-0.9	1.45	211	63	-	-	211	35	214	30
LixiLan-O	iGlarLixi	468	-1.6	0.87	-0.3	4.33	-3.5	2.16	468	345	468	251	468	202	468	149
	Basal insulin	466	-1.3	0.86	1.1	4.32	-3.3	2.16	466	277	466	207	466	117	466	88
	GLP1RA	233	-0.9	0.76	-2.3	4.58	-1.5	1.53	233	77	233	71	233	65	233	61
LixiLan-O-AP	iGlarLixi	348	-1.9	0.75	0.03	2.98	-3.5	1.87	348	275	-	-	348	160	348	153
	Basal insulin	349	-1.4	0.75	1.12	2.99	-3.6	1.87	349	211	-	-	349	279	349	76
	GLP1RA	175	-0.9	0.79	-1.7	4.11	-1.5	1.32	175	52	-	-	175	75	175	43
Soli-D	iGlarLixi	291	-1.88	0.85	-0.3	4.95	-3.2	1.88	291	211	291	194	291	118	291	77
	Biphasic insulin	291	-1.68	0.85	1.19	4.26	-3.5	1.88	291	174	291	163	291	62	291	39
SoliMix	iGlarLixi	443	-1.3	2.1	-0.7	4.2	-1.2	2.4	443	187	443	175	443	141	443	86
	Biphasic insulin	444	-1.1	2.1	1.2	4.2	-0.2	2.59	444	141	444	118	444	351	444	31
VARIATION 2 SA	iGlarLixi	51	-0.8	1.23	1.2	15.36	-1.5	1.77	-	-	-	-	-	-	-	-
	Basal insulin	53	-0.9	1.21	2.8	15.09	-1.1	1.71	-	-	-	-	-	-	-	-
Ji 2025	HR17031	183	-2.4	1.35	-0.1	2.7	-4.1	1.9	183	149	183	104	183	81	183	56
	Basal insulin	182	-1.5	1.34	2	2.7	-3.8	1.35	182	87	182	58	182	24	182	16
	GLP1RA	90	-1.7	0.95	-3.2	3.8	-2.8	1.35	90	50	90	49	90	43	90	42

Abbreviations: BB, basal–bolus; FPG, fasting plasma glucose; GLP-1RA, glucagon-like peptide-1 receptor agonist; HbA1c, glycated hemoglobin; IDegLira, insulin degludec/liraglutide; IcoSema, insulin icodec/semaglutide; iGlarLixi, insulin glargine/lixisenatide; SD, standard deviation.

Supplementary Figure 1. Risk-of-bias assessment of included studies assessed using the Cochrane Risk of Bias 2 tool. (A) Risk-of-bias summary showing review authors' judgments for each risk-of-bias domain in each included study. **(B)** Risk-of-bias graph showing the proportion of included studies judged to be at low risk, some concerns, or high risk of bias for each domain.

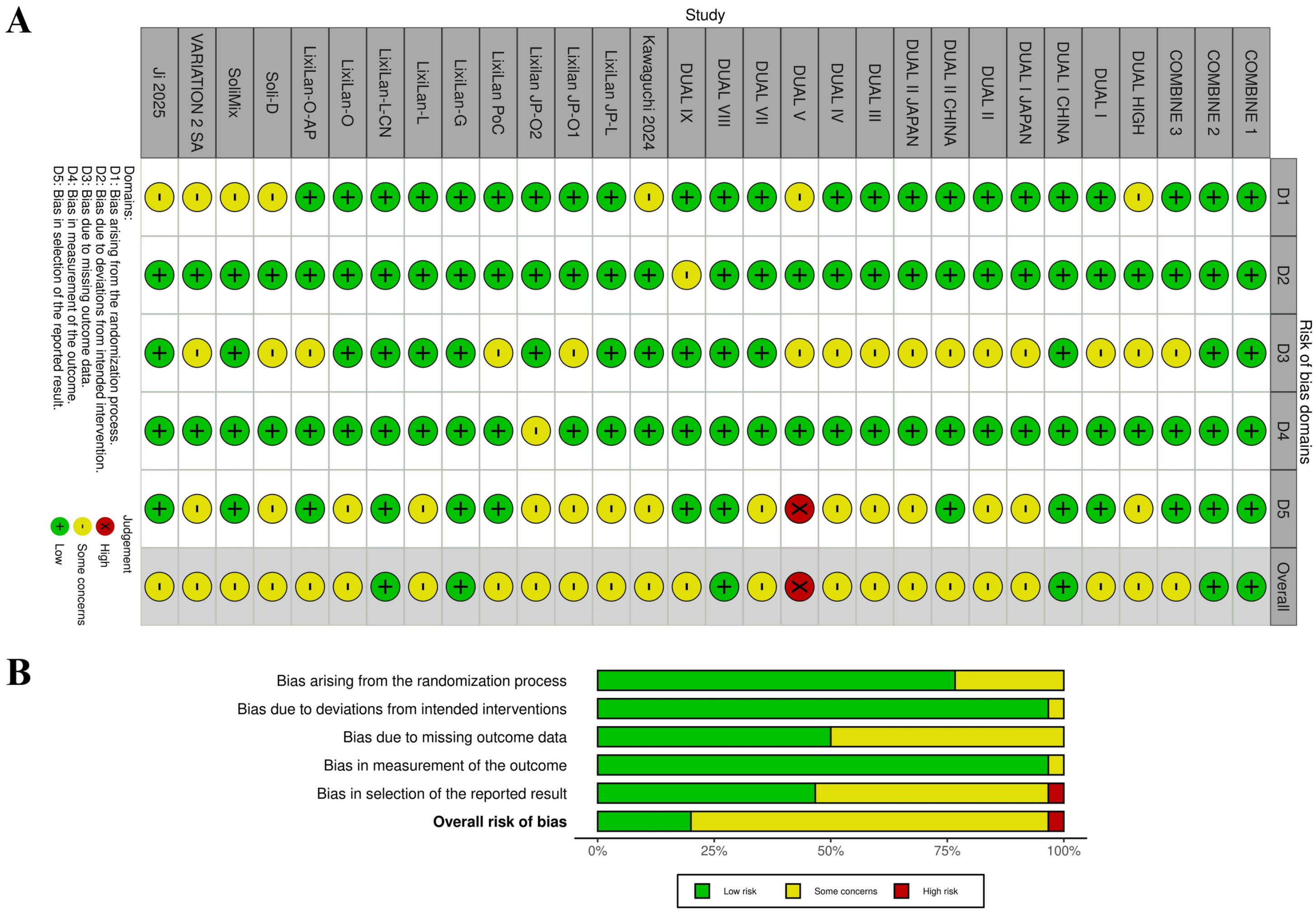

Supplementary Figure 2. Comparison-adjusted funnel plots for the primary continuous outcomes. Funnel plots are shown for change in HbA1c (%) **(A)** and change in body weight (kg) **(B)** Each point represents a study comparison. The plots were used to explore small-study effects and potential publication bias in the network meta-analysis.

A

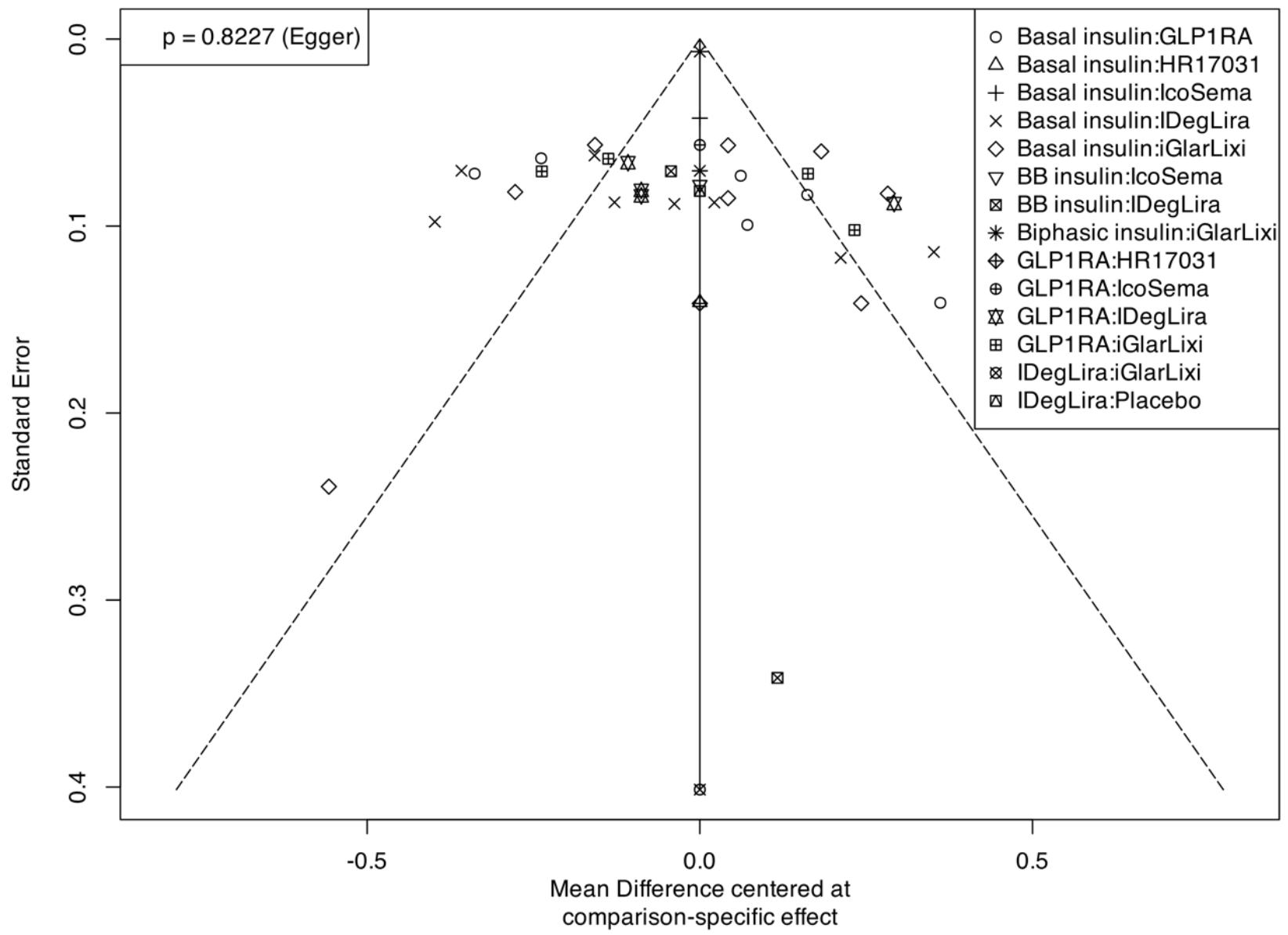

B

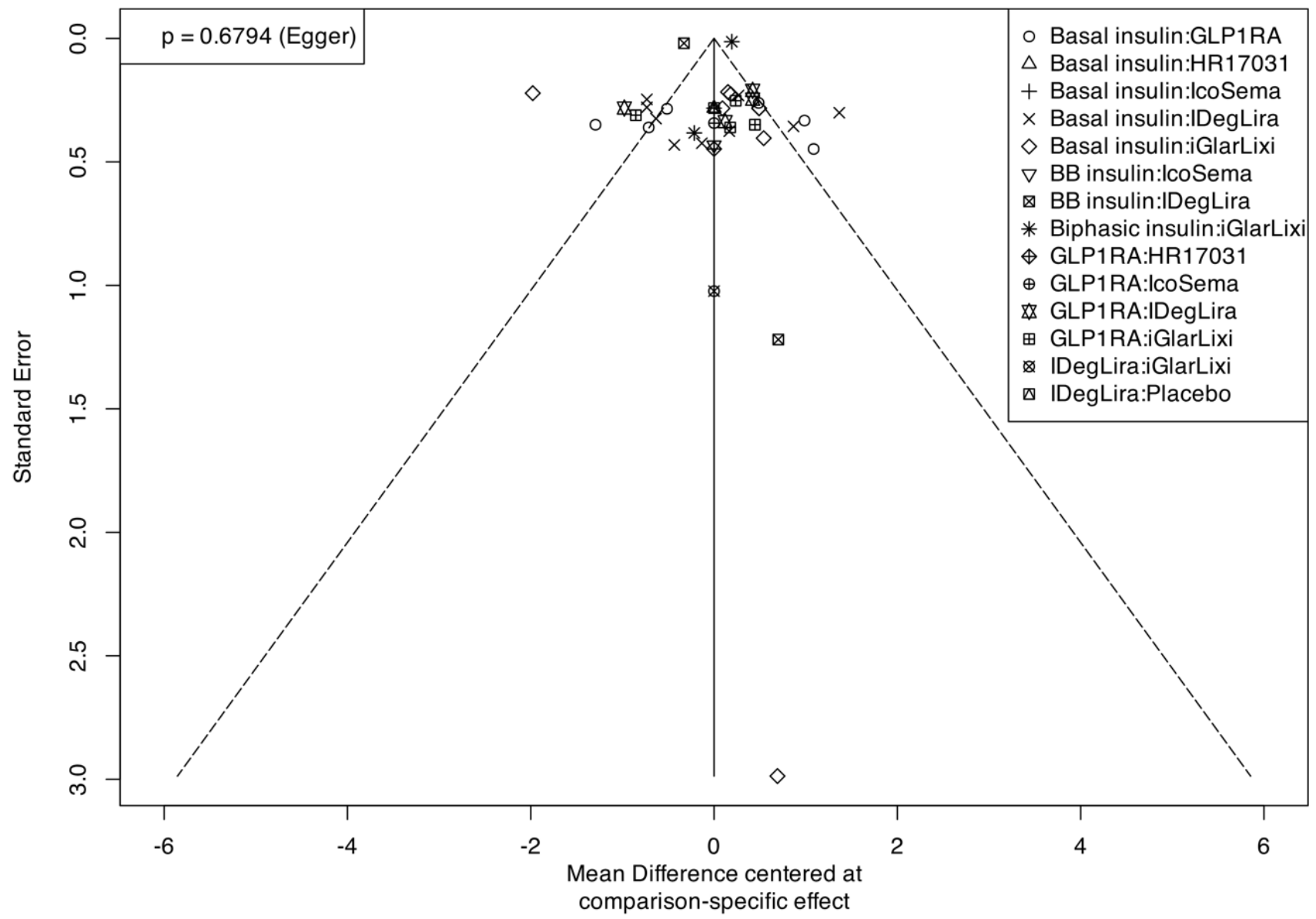

Supplementary Table 3. Network league table for change in HbA1c (%). Results are expressed as MDs with 95% CIs. The lower triangle presents random-effects network meta-analysis estimates for the treatment in the column relative to the treatment in the row; the upper triangle presents direct-comparison estimates for the treatment in the row relative to the treatment in the column. For change in HbA1c, negative MDs indicate greater reduction in the outcome.

**HR17031**	.	.	.	.	.	-0.90 [-1.46; -0.34]	-0.70 [-1.26; -0.14]	.
-0.08 [-0.59; 0.42]	**IDegLira**	-0.10 [-1.02; 0.82]	-0.06 [-0.49; 0.37]	.	.	-0.66 [-0.83; -0.48]	-0.71 [-0.96; -0.45]	-1.00 [-1.51; -0.49]
-0.19 [-0.70; 0.32]	-0.11 [-0.32; 0.11]	**iGlarLixi**	.	.	-0.20 [-0.55; 0.15]	-0.46 [-0.64; -0.27]	-0.84 [-1.09; -0.58]	.
-0.20 [-0.81; 0.41]	-0.12 [-0.47; 0.24]	-0.01 [-0.41; 0.39]	**BB insulin**	0.07 [-0.44; 0.58]	.	.	.	.
-0.21 [-0.78; 0.37]	-0.12 [-0.46; 0.21]	-0.02 [-0.37; 0.33]	-0.01 [-0.39; 0.37]	**IcoSema**	.	-0.66 [-1.15; -0.17]	-0.45 [-0.95; 0.05]	.
-0.39 [-1.01; 0.23]	-0.31 [-0.72; 0.10]	-0.20 [-0.55; 0.15]	-0.19 [-0.72; 0.34]	-0.18 [-0.67; 0.31]	**Biphasic insulin**	.	.	.
-0.72 [-1.21; -0.23]	-0.64 [-0.80; -0.48]	-0.53 [-0.70; -0.37]	-0.52 [-0.89; -0.15]	-0.52 [-0.84; -0.20]	-0.33 [-0.72; 0.05]	**Basal insulin**	-0.16 [-0.37; 0.05]	.
-0.88 [-1.37; -0.39]	-0.80 [-0.99; -0.60]	-0.69 [-0.88; -0.50]	-0.68 [-1.06; -0.30]	-0.67 [-1.00; -0.35]	-0.49 [-0.89; -0.09]	-0.16 [-0.32; 0.01]	**GLP1RA**	.
-1.08 [-1.80; -0.36]	-1.00 [-1.51; -0.49]	-0.89 [-1.45; -0.34]	-0.88 [-1.50; -0.26]	-0.88 [-1.48; -0.27]	-0.69 [-1.35; -0.04]	-0.36 [-0.89; 0.17]	-0.20 [-0.75; 0.34]	**Placebo**

Supplementary Figure 3. Node-splitting analysis for change in HbA1c (%). Separate direct and indirect estimates are presented for each evaluable treatment comparison, together with the corresponding inconsistency assessment. Results are expressed as mean differences (MDs) with 95% confidence intervals (CIs).

Separate indirect from direct evidence (SIDE) using back-calculation method

Random effects model:

comparison	k	prop	nma	direct	indir.	Diff	z	p-value
GLP1RA:Basal insulin	6	0.64	0.1560	0.1615	0.1461	0.0154	0.09	0.9315
HR17031:Basal insulin	1	0.77	-0.7224	-0.9000	-0.1153	-0.7847	-1.31	0.1890
IcoSema:Basal insulin	1	0.42	-0.5152	-0.6600	-0.4083	-0.2517	-0.76	0.4460
IDegLira:Basal insulin	9	0.84	-0.6398	-0.6583	-0.5440	-0.1142	-0.52	0.6030
iGlarLixi:Basal insulin	8	0.81	-0.5340	-0.4575	-0.8538	0.3963	1.84	0.0658
BB insulin:IcoSema	1	0.56	-0.0064	0.0700	-0.1048	0.1748	0.45	0.6553
BB insulin:IDegLira	2	0.69	0.1181	0.0633	0.2381	-0.1748	-0.45	0.6553
GLP1RA:HR17031	1	0.77	0.8783	0.7000	1.4831	-0.7831	-1.31	0.1890
GLP1RA:IcoSema	1	0.43	0.6712	0.4500	0.8359	-0.3859	-1.15	0.2488
GLP1RA:IDegLira	4	0.57	0.7957	0.7080	0.9127	-0.2047	-1.03	0.3017
GLP1RA:iGlarLixi	4	0.58	0.6900	0.8375	0.4822	0.3553	1.77	0.0771
IDegLira:iGlarLixi	1	0.05	-0.1057	-0.1000	-0.1060	0.0060	0.01	0.9901

Legend:
comparison - Treatment comparison
k - Number of studies providing direct evidence
prop - Direct evidence proportion
nma - Estimated treatment effect (MD) in network meta-analysis
direct - Estimated treatment effect (MD) derived from direct evidence
indir. - Estimated treatment effect (MD) derived from indirect evidence
Diff - Difference between direct and indirect treatment estimates
z - z-value of test for disagreement (direct versus indirect)
p-value - p-value of test for disagreement (direct versus indirect)

Supplementary Table 4. Network league table for the change in body weight (kg). Results are expressed as MDs with 95% CIs. The lower triangle presents random-effects network meta-analysis estimates for the treatment in the column relative to the treatment in the row; the upper triangle presents direct-comparison estimates for the treatment in the row relative to the treatment in the column. For change in body weight, negative MDs indicate greater reduction in the outcome.

**GLP1RA**	-4.54 [-6.88; -2.20]	.	-3.10 [-5.51; -0.69]	-2.92 [-4.07; -1.77]	-2.18 [-3.34; -1.01]	-4.11 [-5.07; -3.16]	.	.
-1.26 [-2.78; 0.25]	**IcoSema**	.	.	.	.	-5.59 [-7.90; -3.28]	.	-6.72 [-9.12; -4.32]
-1.27 [-3.74; 1.20]	-0.01 [-2.78; 2.77]	**Placebo**	.	-1.50 [-3.81; 0.81]	.	.	.	.
-2.68 [-4.75; -0.61]	-1.42 [-3.91; 1.07]	-1.41 [-4.56; 1.74]	**HR17031**	.	.	-2.10 [-4.41; 0.21]	.	.
-2.77 [-3.64; -1.91]	-1.51 [-3.05; 0.03]	-1.50 [-3.81; 0.81]	-0.09 [-2.23; 2.05]	**IDegLira**	0.70 [-2.31; 3.71]	-1.83 [-2.61; -1.05]	.	-3.90 [-5.75; -2.05]
-3.05 [-3.94; -2.17]	-1.79 [-3.41; -0.17]	-1.78 [-4.28; 0.72]	-0.37 [-2.52; 1.78]	-0.28 [-1.24; 0.68]	**iGlarLixi**	-0.91 [-1.77; -0.04]	-1.71 [-3.33; -0.08]	.
-4.42 [-5.19; -3.66]	-3.16 [-4.66; -1.67]	-3.15 [-5.57; -0.74]	-1.74 [-3.80; 0.31]	-1.65 [-2.36; -0.95]	-1.37 [-2.13; -0.61]	**Basal insulin**	.	.
-4.76 [-6.61; -2.91]	-3.50 [-5.79; -1.20]	-3.49 [-6.47; -0.50]	-2.08 [-4.78; 0.62]	-1.99 [-3.88; -0.10]	-1.71 [-3.33; -0.08]	-0.33 [-2.13; 1.46]	**Biphasic insulin**	.
-7.16 [-8.87; -5.45]	-5.90 [-7.65; -4.14]	-5.89 [-8.68; -3.09]	-4.48 [-7.08; -1.87]	-4.39 [-5.96; -2.82]	-4.11 [-5.88; -2.33]	-2.73 [-4.39; -1.08]	-2.40 [-4.81; 0.01]	**BB insulin**

Supplementary Figure 4. Node-splitting analysis for change in body weight (kg). Separate direct and indirect estimates are presented for each evaluable treatment comparison, together with the corresponding inconsistency assessment. Results are expressed as MDs with 95% CIs.

```
Separate indirect from direct evidence (SIDE) using back-calculation method

Random effects model:

              comparison k prop     nma  direct  indir.    Diff     z p-value
    GLP1RA:Basal insulin 6 0.65 -4.4250 -4.1132 -4.9955  0.8823  1.08  0.2821
   HR17031:Basal insulin 1 0.79 -1.7434 -2.1000 -0.3745 -1.7255 -0.67  0.5056
   IcoSema:Basal insulin 1 0.42 -3.1627 -5.5900 -1.4205 -4.1695 -2.70  0.0069
  IDegLira:Basal insulin 9 0.83 -1.6539 -1.8333 -0.8011 -1.0322 -1.08  0.2788
 iGlarLixi:Basal insulin 8 0.78 -1.3724 -0.9080 -3.0015  2.0935  2.24  0.0253
      BB insulin:IcoSema 1 0.54  5.8976  6.7200  4.9493  1.7707  0.99  0.3237
     BB insulin:IDegLira 2 0.72  4.3889  3.8985  5.6692 -1.7707 -0.99  0.3237
          GLP1RA:HR17031 1 0.74 -2.6816 -3.1000 -1.5019 -1.5981 -0.67  0.5056
          GLP1RA:IcoSema 1 0.42 -1.2623 -4.5400  1.0938 -5.6338 -3.60  0.0003
         GLP1RA:IDegLira 4 0.56 -2.7711 -2.9215 -2.5766 -0.3450 -0.39  0.6985
        GLP1RA:iGlarLixi 4 0.57 -3.0526 -2.1766 -4.2374  2.0608  2.26  0.0237
      IDegLira:iGlarLixi 1 0.10 -0.2815  0.7000 -0.3921  1.0921  0.67  0.5000

Legend:
 comparison - Treatment comparison
 k          - Number of studies providing direct evidence
 prop       - Direct evidence proportion
 nma        - Estimated treatment effect (MD) in network meta-analysis
 direct     - Estimated treatment effect (MD) derived from direct evidence
 indir.     - Estimated treatment effect (MD) derived from indirect evidence
 Diff       - Difference between direct and indirect treatment estimates
 z          - z-value of test for disagreement (direct versus indirect)
 p-value    - p-value of test for disagreement (direct versus indirect)
```

Supplementary Figure 5. Network meta-analysis for change in fasting plasma glucose (mmol/L). (A) Network plot of treatment comparisons. Node size is proportional to the number of participants receiving the respective intervention; line thickness is proportional to the number of studies contributing direct evidence, and numbers on lines indicate the number of studies. **(B)** Random-effects network meta-analysis forest plot showing mean differences (MDs) with 95% confidence intervals (CIs) for change in fasting plasma glucose, with basal insulin as the reference treatment. Negative MDs favor greater fasting plasma glucose reduction. **(C)** P-scores for change in fasting plasma glucose. **(D)** Node-splitting analysis comparing direct and indirect evidence using the back-calculation method.

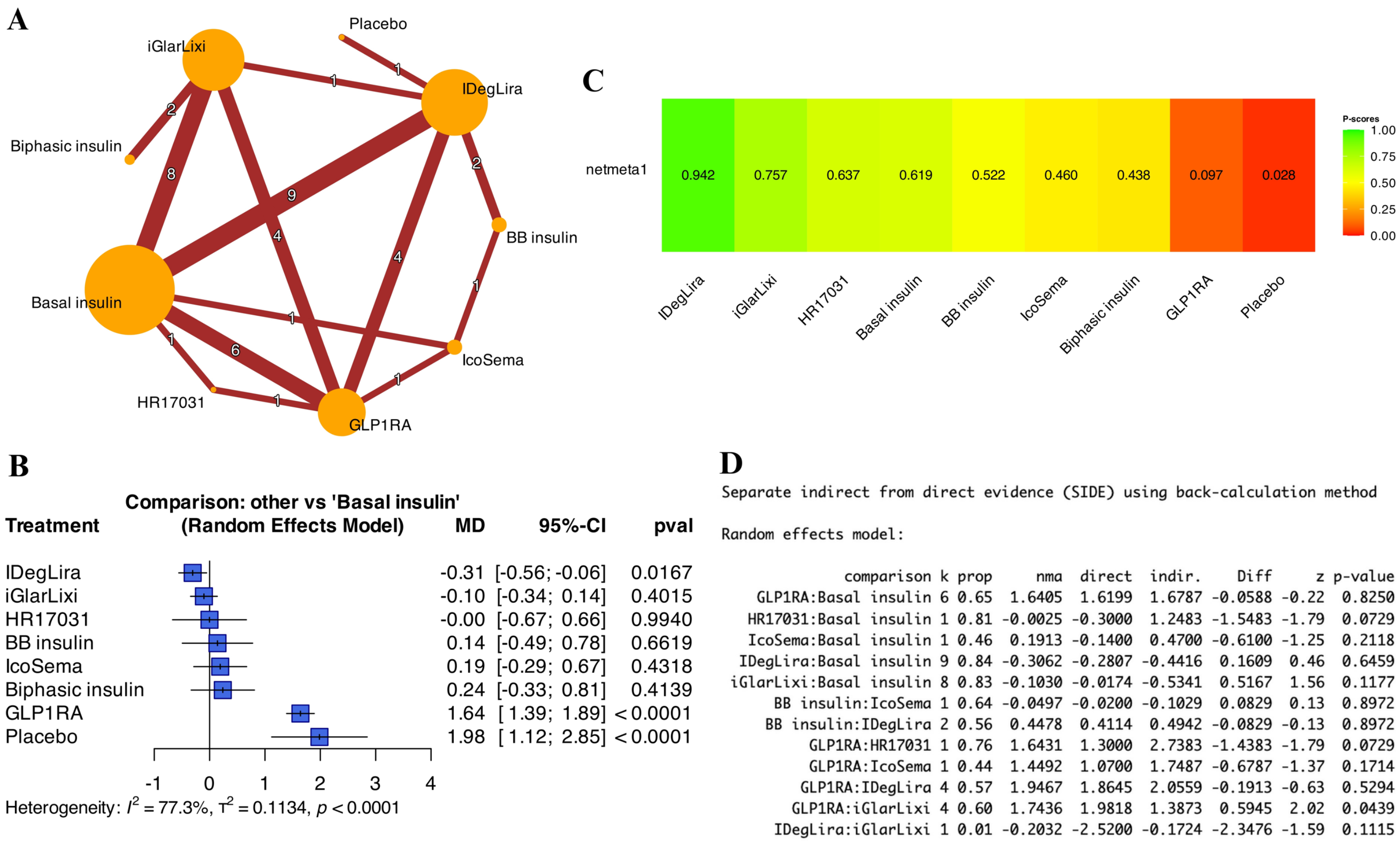

Supplementary Table 5. Network league table for the change in fasting plasma glucose (mmol/L). Results are expressed as MDs with 95% CIs. The lower triangle presents random-effects network meta-analysis estimates for the treatment in the column relative to the treatment in the row; the upper triangle presents direct-comparison estimates for the treatment in the row relative to the treatment in the column. For change in fasting plasma glucose, negative MDs indicate greater reduction in the outcome.

**IDegLira**	-2.52 [-5.39; 0.35]	.	-0.28 [-0.55; -0.01]	-0.41 [-1.25; 0.42]	.	.	-1.86 [-2.26; -1.47]	-2.29 [-3.12; -1.46]
-0.20 [-0.53; 0.13]	**iGlarLixi**	.	-0.02 [-0.28; 0.25]	.	.	-0.34 [-0.86; 0.18]	-1.98 [-2.35; -1.62]	.
-0.30 [-1.00; 0.40]	-0.10 [-0.80; 0.60]	**HR17031**	-0.30 [-1.04; 0.44]	.	.	.	-1.30 [-2.07; -0.53]	.
-0.31 [-0.56; -0.06]	-0.10 [-0.34; 0.14]	-0.00 [-0.67; 0.66]	**Basal insulin**	.	0.14 [-0.57; 0.85]	.	-1.62 [-1.93; -1.31]	.
-0.45 [-1.07; 0.18]	-0.24 [-0.91; 0.42]	-0.14 [-1.05; 0.76]	-0.14 [-0.78; 0.49]	**BB insulin**	-0.02 [-0.77; 0.73]	.	.	.
-0.50 [-1.00; 0.01]	-0.29 [-0.81; 0.22]	-0.19 [-1.00; 0.61]	-0.19 [-0.67; 0.29]	-0.05 [-0.65; 0.55]	**IcoSema**	.	-1.07 [-1.80; -0.34]	.
-0.54 [-1.16; 0.07]	-0.34 [-0.86; 0.18]	-0.24 [-1.11; 0.63]	-0.24 [-0.81; 0.33]	-0.10 [-0.94; 0.75]	-0.05 [-0.78; 0.69]	**Biphasic insulin**	.	.
-1.95 [-2.24; -1.65]	-1.74 [-2.03; -1.46]	-1.64 [-2.31; -0.97]	-1.64 [-1.89; -1.39]	-1.50 [-2.14; -0.85]	-1.45 [-1.93; -0.97]	-1.40 [-1.99; -0.81]	**GLP1RA**	.
-2.29 [-3.12; -1.46]	-2.09 [-2.98; -1.20]	-1.99 [-3.07; -0.90]	-1.98 [-2.85; -1.12]	-1.84 [-2.88; -0.81]	-1.79 [-2.76; -0.83]	-1.75 [-2.77; -0.72]	-0.34 [-1.22; 0.53]	**Placebo**

Supplementary Figure 6. Network meta-analysis for the proportion of participants achieving HbA1c <7%. (A) Network plot of treatment comparisons. Node size is proportional to the number of participants receiving the respective intervention; line thickness is proportional to the number of studies contributing direct evidence, and numbers on lines indicate the number of studies. **(B)** Random-effects network meta-analysis forest plot showing odds ratios (ORs) with 95% CIs for achievement of HbA1c <7%, with basal insulin as the reference treatment. ORs greater than 1 favor the intervention over basal insulin. **(C)** P-scores for achievement of HbA1c <7%. **(D)** Node-splitting analysis comparing direct and indirect evidence using the back-calculation method.

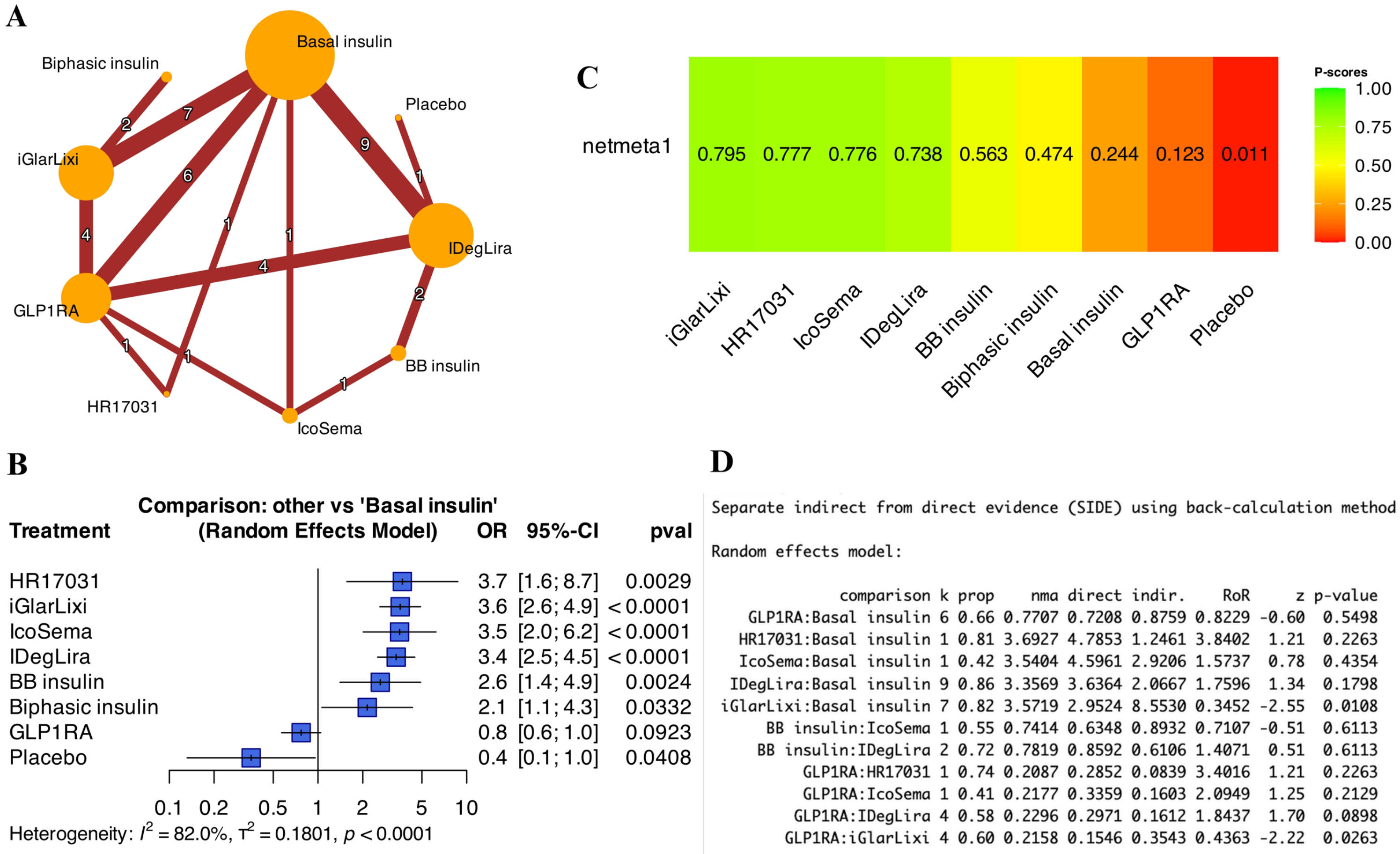

Separate indirect from direct evidence (SIDE) using back-calculation method

Random effects model:

comparison	k	prop	nma	direct	indir.	RoR	z	p-value
GLP1RA:Basal insulin	6	0.66	0.7707	0.7208	0.8759	0.8229	-0.60	0.5498
HR17031:Basal insulin	1	0.81	3.6927	4.7853	1.2461	3.8402	1.21	0.2263
IcoSema:Basal insulin	1	0.42	3.5404	4.5961	2.9206	1.5737	0.78	0.4354
IDegLira:Basal insulin	9	0.86	3.3569	3.6364	2.0667	1.7596	1.34	0.1798
iGlarLixi:Basal insulin	7	0.82	3.5719	2.9524	8.5530	0.3452	-2.55	0.0108
BB insulin:IcoSema	1	0.55	0.7414	0.6348	0.8932	0.7107	-0.51	0.6113
BB insulin:IDegLira	2	0.72	0.7819	0.8592	0.6106	1.4071	0.51	0.6113
GLP1RA:HR17031	1	0.74	0.2087	0.2852	0.0839	3.4016	1.21	0.2263
GLP1RA:IcoSema	1	0.41	0.2177	0.3359	0.1603	2.0949	1.25	0.2129
GLP1RA:IDegLira	4	0.58	0.2296	0.2971	0.1612	1.8437	1.70	0.0898
GLP1RA:iGlarLixi	4	0.60	0.2158	0.1546	0.3543	0.4363	-2.22	0.0263

Supplementary Table 6. Network league table for the proportions of study subjects who achieved HbA1c <7%. Results are expressed as odds ratios with 95% CIs. The lower triangle presents random-effects network meta-analysis estimates for the treatment in the column relative to the treatment in the row; the upper triangle presents direct-comparison estimates for the treatment in the row relative to the treatment in the column. Values greater than 1 favor the treatment in the numerator for achievement of the specified endpoint.

**iGlarLixi**	.	.	.	.	1.7 [0.9; 3.1]	3.0 [2.1; 4.2]	6.5 [4.1; 10.3]	.
1.0 [0.4; 2.4]	**HR17031**	.	.	.	.	4.8 [1.8; 12.5]	3.5 [1.3; 9.5]	.
1.0 [0.5; 1.9]	1.0 [0.4; 2.9]	**IcoSema**	.	1.6 [0.6; 3.8]	.	4.6 [1.9; 10.9]	3.0 [1.2; 7.3]	.
1.1 [0.7; 1.6]	1.1 [0.5; 2.7]	1.1 [0.6; 1.9]	**IDegLira**	1.2 [0.6; 2.3]	.	3.6 [2.7; 5.0]	3.4 [2.1; 5.3]	9.5 [3.7; 24.4]
1.4 [0.7; 2.7]	1.4 [0.5; 4.0]	1.3 [0.7; 2.6]	1.3 [0.7; 2.3]	**BB insulin**	.	.	.	.
1.7 [0.9; 3.1]	1.7 [0.6; 5.2]	1.7 [0.7; 4.0]	1.6 [0.7; 3.3]	1.2 [0.5; 3.1]	**Biphasic insulin**	.	.	.
3.6 [2.6; 4.9]	3.7 [1.6; 8.7]	3.5 [2.0; 6.2]	3.4 [2.5; 4.5]	2.6 [1.4; 4.9]	2.1 [1.1; 4.3]	**Basal insulin**	1.4 [1.0; 2.0]	.
4.6 [3.2; 6.6]	4.8 [2.0; 11.4]	4.6 [2.6; 8.1]	4.4 [3.1; 6.2]	3.4 [1.8; 6.5]	2.8 [1.3; 5.7]	1.3 [1.0; 1.8]	**GLP1RA**	.
10.1 [3.6; 28.2]	10.4 [2.8; 38.3]	10.0 [3.3; 30.4]	9.5 [3.7; 24.4]	7.4 [2.4; 22.6]	6.0 [1.8; 20.2]	2.8 [1.0; 7.6]	2.2 [0.8; 6.0]	**Placebo**

Supplementary Figure 7. Network meta-analysis for the proportion of participants achieving HbA1c <7% without weight gain. (A) Network plot of treatment comparisons. Node size is proportional to the number of participants receiving the respective intervention; line thickness is proportional to the number of studies contributing direct evidence, and numbers on lines indicate the number of studies. **(B)** Random-effects network meta-analysis forest plot showing ORs with 95% CIs for achievement of HbA1c <7% without weight gain, with basal insulin as the reference treatment. ORs greater than 1 favor the intervention over basal insulin. **(C)** P-scores for achievement of HbA1c <7% without weight gain. **(D)** Node-splitting analysis comparing direct and indirect evidence using the back-calculation method.

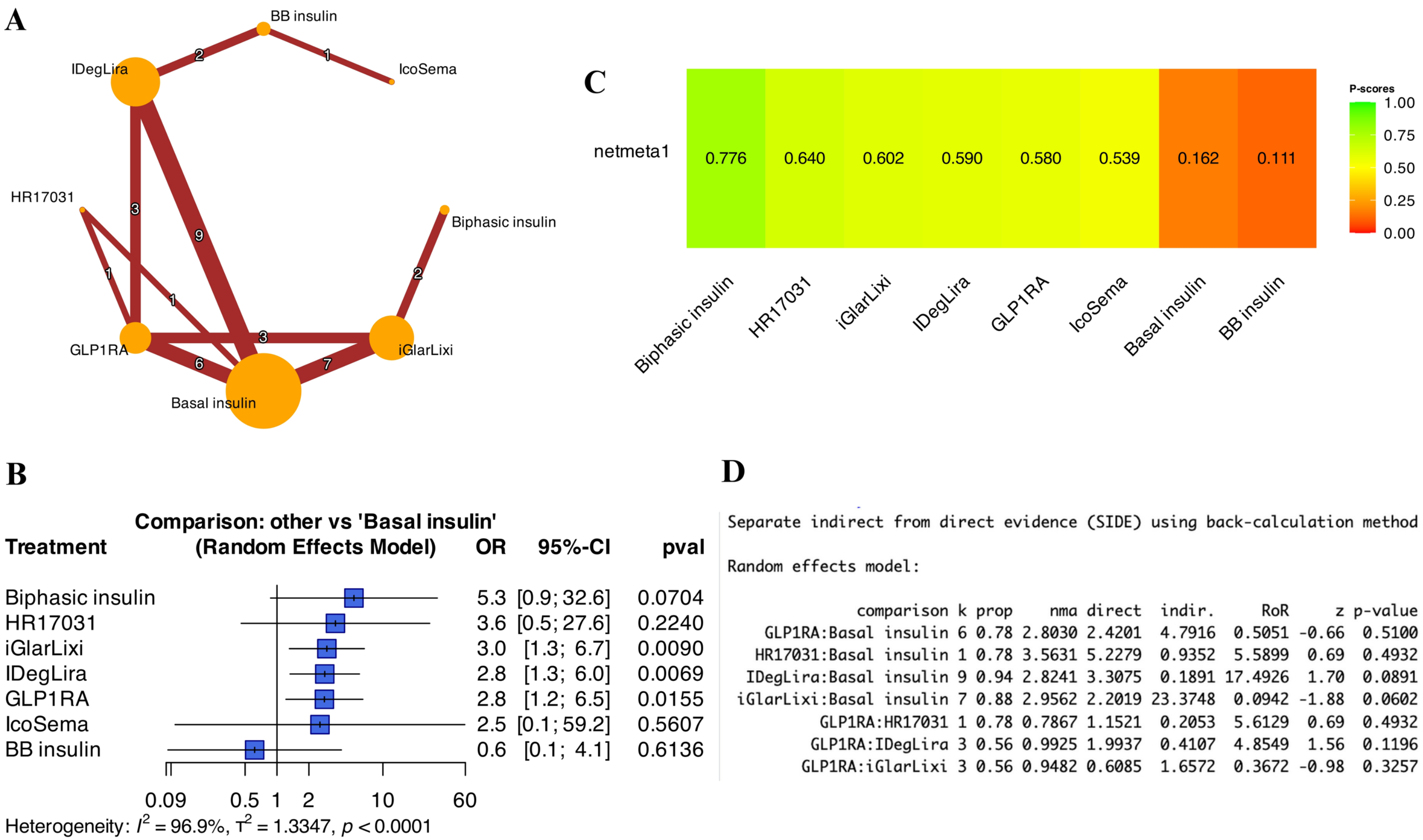

Separate indirect from direct evidence (SIDE) using back-calculation method

Random effects model:

comparison	k	prop	nma	direct	indir.	RoR	z	p-value
GLP1RA:Basal insulin	6	0.78	2.8030	2.4201	4.7916	0.5051	-0.66	0.5100
HR17031:Basal insulin	1	0.78	3.5631	5.2279	0.9352	5.5899	0.69	0.4932
IDegLira:Basal insulin	9	0.94	2.8241	3.3075	0.1891	17.4926	1.70	0.0891
iGlarLixi:Basal insulin	7	0.88	2.9562	2.2019	23.3748	0.0942	-1.88	0.0602
GLP1RA:HR17031	1	0.78	0.7867	1.1521	0.2053	5.6129	0.69	0.4932
GLP1RA:IDegLira	3	0.56	0.9925	1.9937	0.4107	4.8549	1.56	0.1196
GLP1RA:iGlarLixi	3	0.56	0.9482	0.6085	1.6572	0.3672	-0.98	0.3257

Supplementary Table 7. Network league table for the proportions of study subjects who achieved HbA1c <7% without weight gain. Results are expressed as odds ratios with 95% CIs. The lower triangle presents random-effects network meta-analysis estimates for the treatment in the column relative to the treatment in the row; the upper triangle presents direct-comparison estimates for the treatment in the row relative to the treatment in the column. Values greater than 1 favor the treatment in the numerator for achievement of the specified endpoint.

**Biphasic insulin**	.	1.8 [0.4; 9.1]	.	.	.	.	.
1.5 [0.1; 22.2]	**HR17031**	.	.	0.9 [0.1; 8.8]	.	5.2 [0.5; 53.4]	.
1.8 [0.4; 9.1]	1.2 [0.1; 10.5]	**iGlarLixi**	.	1.6 [0.4; 6.2]	.	2.2 [0.9; 5.3]	.
1.9 [0.3; 13.2]	1.3 [0.1; 10.8]	1.0 [0.4; 3.1]	**IDegLira**	0.5 [0.1; 1.9]	.	3.3 [1.5; 7.2]	4.6 [0.8; 25.8]
1.9 [0.3; 12.7]	1.3 [0.2; 9.9]	1.1 [0.4; 2.8]	1.0 [0.4; 2.7]	**GLP1RA**	.	2.4 [0.9; 6.2]	.
2.1 [0.1; 78.2]	1.4 [0.0; 58.7]	1.2 [0.0; 29.6]	1.1 [0.1; 23.6]	1.1 [0.0; 27.3]	**IcoSema**	.	4.1 [0.3; 51.4]
5.3 [0.9; 32.6]	3.6 [0.5; 27.6]	3.0 [1.3; 6.7]	2.8 [1.3; 6.0]	2.8 [1.2; 6.5]	2.5 [0.1; 59.2]	**Basal insulin**	.
8.7 [0.6; 116.5]	5.8 [0.4; 91.2]	4.8 [0.6; 36.7]	4.6 [0.8; 25.8]	4.6 [0.6; 33.3]	4.1 [0.3; 51.4]	1.6 [0.2; 10.7]	**BB insulin**

Supplementary Figure 8. Network meta-analysis for the proportion of participants achieving HbA1c <7% without clinically significant or severe hypoglycemic episodes. (A) Network plot of treatment comparisons. Node size is proportional to the number of participants receiving the respective intervention; line thickness is proportional to the number of studies contributing direct evidence, and numbers on lines indicate the number of studies. **(B)** Random-effects network meta-analysis forest plot showing ORs with 95% CIs for achievement of HbA1c <7% without clinically significant or severe hypoglycemic episodes, with basal insulin as the reference treatment. ORs greater than 1 favor the intervention over basal insulin. **(C)** P-scores for achievement of HbA1c <7% without clinically significant or severe hypoglycemic episodes. **(D)** Node-splitting analysis comparing direct and indirect evidence using the back-calculation method.

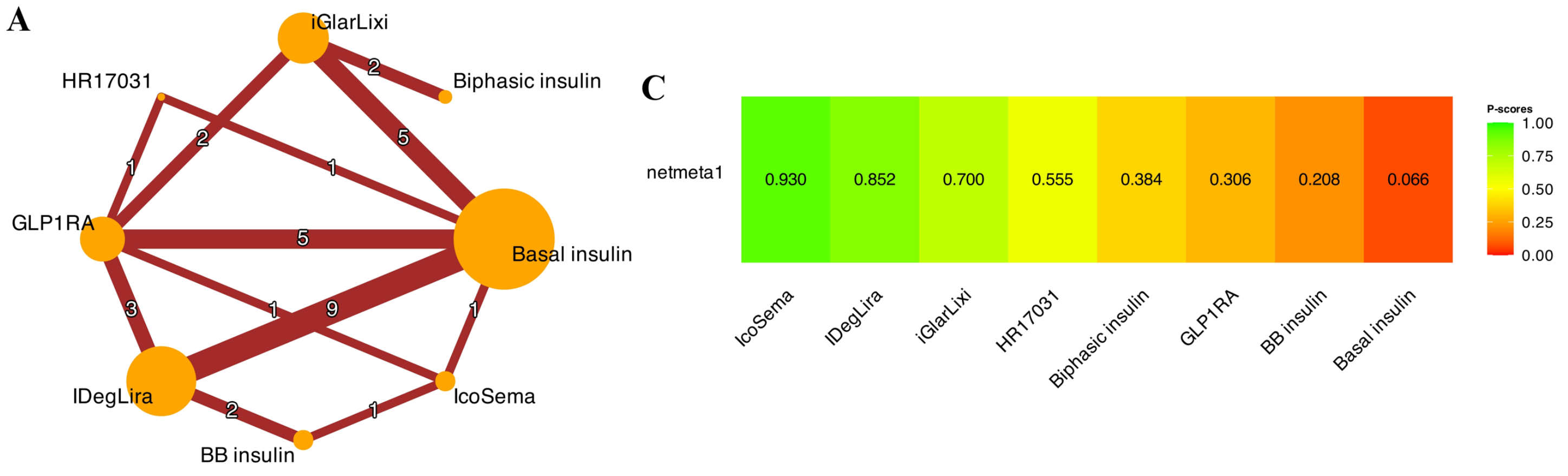

B

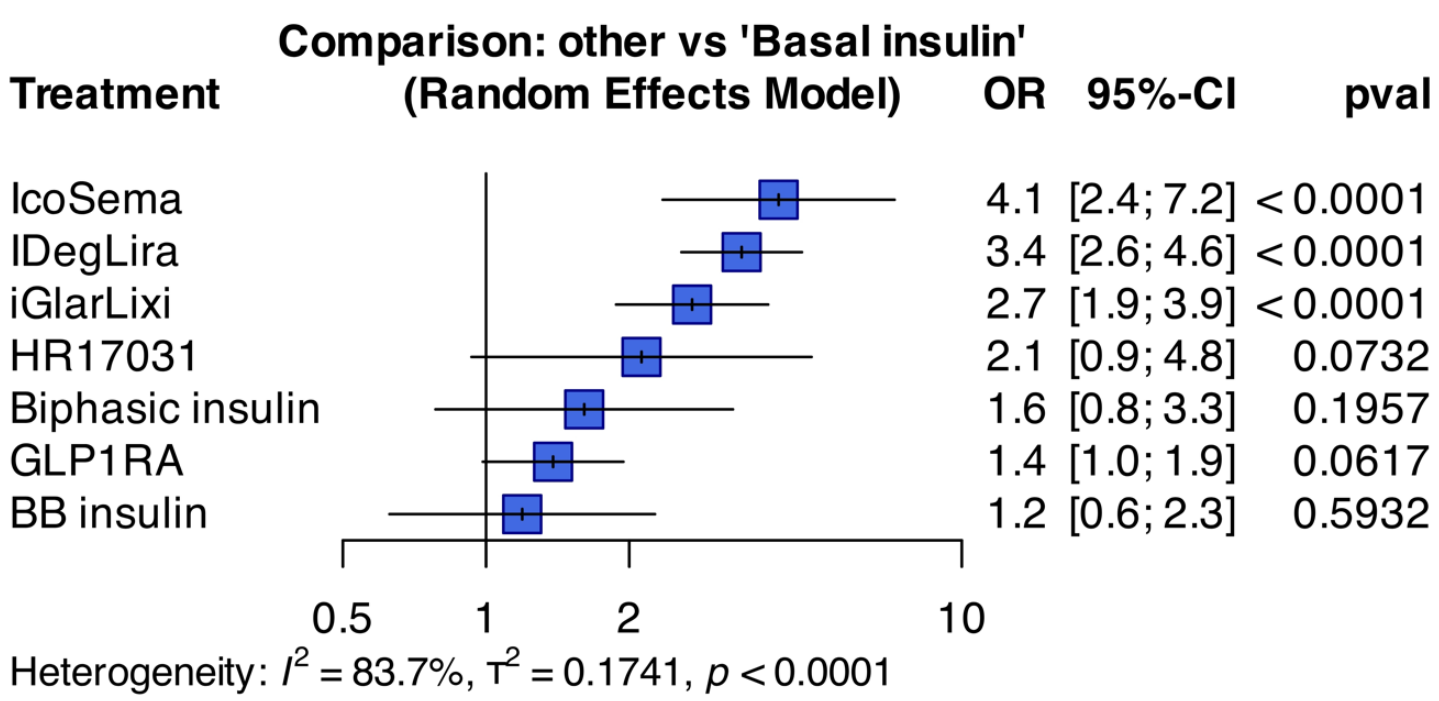

D

Separate indirect from direct evidence (SIDE) using back-calculation method

Random effects model:

comparison	k	prop	nma	direct	indir.	RoR	z	p-value
GLP1RA:Basal insulin	5	0.71	1.3833	1.3783	1.3953	0.9878	-0.03	0.9744
HR17031:Basal insulin	1	0.80	2.1222	2.8145	0.7071	3.9801	1.33	0.1847
IcoSema:Basal insulin	1	0.44	4.1209	4.4976	3.8523	1.1675	0.27	0.7886
IDegLira:Basal insulin	9	0.91	3.4460	3.6047	2.1387	1.6854	0.98	0.3250
iGlarLixi:Basal insulin	5	0.84	2.7112	2.1792	8.4930	0.2566	-2.66	0.0078
BB insulin:IcoSema	1	0.57	0.2891	0.3144	0.2591	1.2133	0.28	0.7758
BB insulin:IDegLira	2	0.70	0.3457	0.3260	0.3955	0.8242	-0.28	0.7758
GLP1RA:HR17031	1	0.74	0.6518	0.9078	0.2523	3.5988	1.33	0.1847
GLP1RA:IcoSema	1	0.43	0.3357	0.3387	0.3334	1.0159	0.03	0.9789
GLP1RA:IDegLira	3	0.57	0.4014	0.5638	0.2582	2.1834	1.96	0.0495
GLP1RA:iGlarLixi	2	0.48	0.5102	0.3139	0.7964	0.3942	-2.09	0.0366

Supplementary Table 8. Network league table for the proportions of study subjects who achieved HbA1c <7% without clinically significant or severe hypoglycemic episodes. Results are expressed as odds ratios with 95% CIs. The lower triangle presents random-effects network meta-analysis estimates for the treatment in the column relative to the treatment in the row; the upper triangle presents direct-comparison estimates for the treatment in the row relative to the treatment in the column. Values greater than 1 favor the treatment in the numerator for achievement of the specified endpoint.

**IcoSema**	.	.	.	.	3.0 [1.2; 7.1]	3.2 [1.3; 7.6]	4.5 [1.9; 10.5]
1.2 [0.7; 2.1]	**IDegLira**	.	.	.	1.8 [1.1; 3.0]	3.1 [1.5; 6.4]	3.6 [2.7; 4.9]
1.5 [0.8; 2.9]	1.3 [0.8; 2.0]	**iGlarLixi**	.	1.7 [0.9; 3.1]	3.2 [1.7; 6.0]	.	2.2 [1.5; 3.3]
1.9 [0.7; 5.1]	1.6 [0.7; 3.8]	1.3 [0.5; 3.1]	**HR17031**	.	1.1 [0.4; 2.9]	.	2.8 [1.1; 7.1]
2.6 [1.0; 6.3]	2.1 [1.0; 4.6]	1.7 [0.9; 3.1]	1.3 [0.4; 3.9]	**Biphasic insulin**	.	.	.
3.0 [1.7; 5.3]	2.5 [1.7; 3.7]	2.0 [1.3; 3.0]	1.5 [0.7; 3.5]	1.2 [0.5; 2.5]	**GLP1RA**	.	1.4 [0.9; 2.1]
3.5 [1.8; 6.7]	2.9 [1.6; 5.3]	2.3 [1.1; 4.7]	1.8 [0.6; 5.0]	1.4 [0.5; 3.5]	1.2 [0.6; 2.3]	**BB insulin**	.
4.1 [2.4; 7.2]	3.4 [2.6; 4.6]	2.7 [1.9; 3.9]	2.1 [0.9; 4.8]	1.6 [0.8; 3.3]	1.4 [1.0; 1.9]	1.2 [0.6; 2.3]	**Basal insulin**

Supplementary Figure 9. Network meta-analysis for the proportion of participants achieving HbA1c <7% without weight gain and without clinically significant or severe hypoglycemic episodes. (A) Network plot of treatment comparisons. Node size is proportional to the number of participants receiving the respective intervention; line thickness is proportional to the number of studies contributing direct evidence; and numbers on lines indicate the number of studies. **(B)** Random-effects network meta-analysis forest plot showing ORs with 95% CIs for achievement of HbA1c <7% without weight gain and without clinically significant or severe hypoglycemic episodes, with basal insulin as the reference treatment. ORs greater than 1 favor the intervention over basal insulin. **(C)** P-scores for achievement of HbA1c <7% without weight gain and without clinically significant or severe hypoglycemic episodes. **(D)** Node-splitting analysis comparing direct and indirect evidence using the back-calculation method.

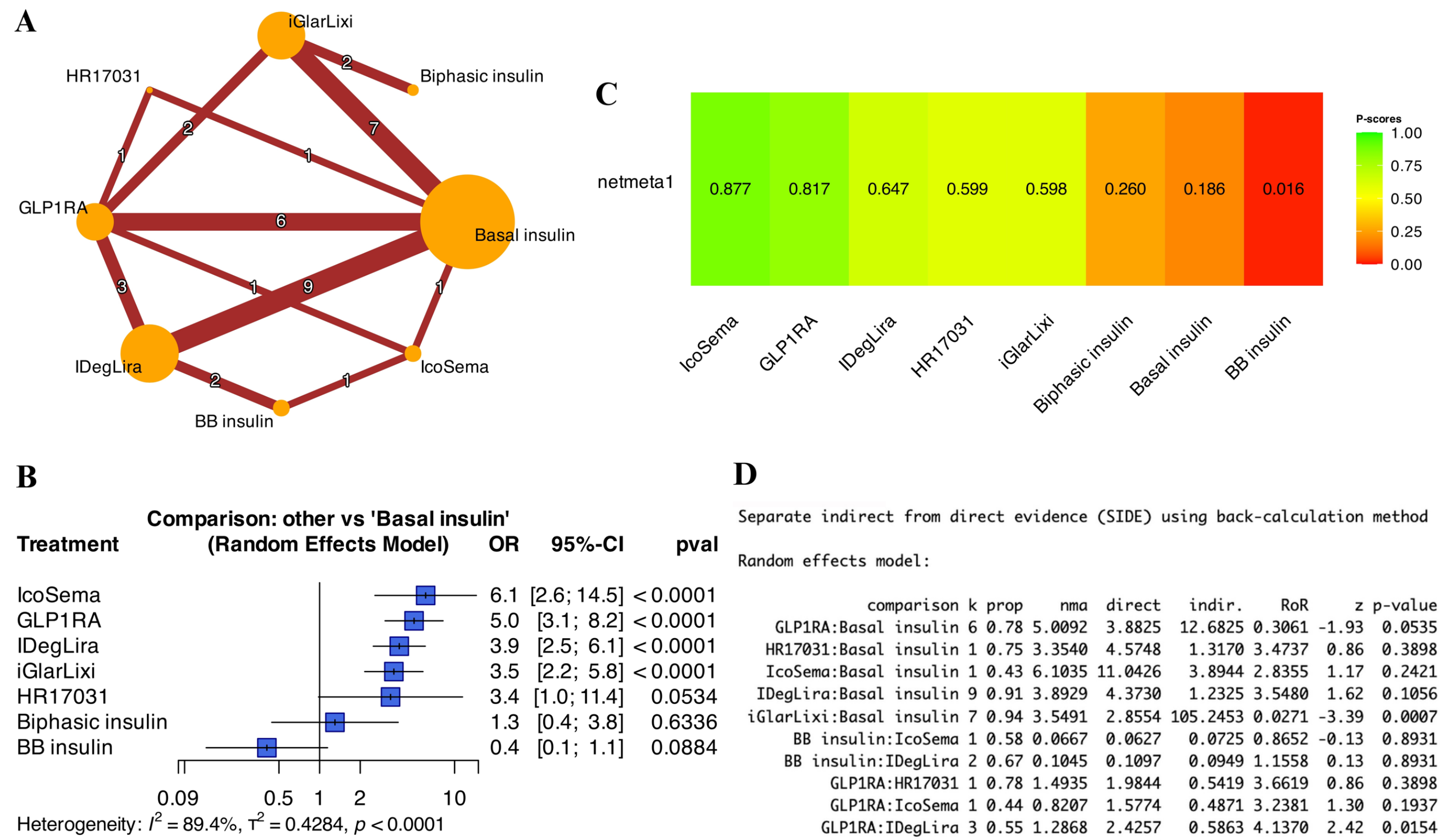

Separate indirect from direct evidence (SIDE) using back-calculation method

Random effects model:

comparison	k	prop	nma	direct	indir.	RoR	z	p-value
GLP1RA:Basal insulin	6	0.78	5.0092	3.8825	12.6825	0.3061	-1.93	0.0535
HR17031:Basal insulin	1	0.75	3.3540	4.5748	1.3170	3.4737	0.86	0.3898
IcoSema:Basal insulin	1	0.43	6.1035	11.0426	3.8944	2.8355	1.17	0.2421
IDegLira:Basal insulin	9	0.91	3.8929	4.3730	1.2325	3.5480	1.62	0.1056
iGlarLixi:Basal insulin	7	0.94	3.5491	2.8554	105.2453	0.0271	-3.39	0.0007
BB insulin:IcoSema	1	0.58	0.0667	0.0627	0.0725	0.8652	-0.13	0.8931
BB insulin:IDegLira	2	0.67	0.1045	0.1097	0.0949	1.1558	0.13	0.8931
GLP1RA:HR17031	1	0.78	1.4935	1.9844	0.5419	3.6619	0.86	0.3898
GLP1RA:IcoSema	1	0.44	0.8207	1.5774	0.4871	3.2381	1.30	0.1937
GLP1RA:IDegLira	3	0.55	1.2868	2.4257	0.5863	4.1370	2.42	0.0154
GLP1RA:iGlarLixi	2	0.45	1.4114	0.5634	3.0074	0.1873	-2.57	0.0102

Supplementary Table 9. Network league table for the proportions of study subjects who achieved HbA1c <7% without weight gain and clinically significant or severe hypoglycemic episodes. Results are expressed as odds ratios with 95% CIs. The lower triangle presents random-effects network meta-analysis estimates for the treatment in the column relative to the treatment in the row; the upper triangle presents direct-comparison estimates for the treatment in the row relative to the treatment in the column. Values greater than 1 favor the treatment in the numerator for achievement of the specified endpoint.

**IcoSema**	0.6 [0.2; 2.4]	.	.	.	.	11.0 [3.0; 41.2]	15.9 [4.0; 63.2]
1.2 [0.5; 2.9]	**GLP1RA**	2.4 [1.1; 5.2]	2.0 [0.5; 7.9]	0.6 [0.2; 1.5]	.	3.9 [2.2; 6.8]	.
1.6 [0.6; 3.9]	1.3 [0.7; 2.3]	**IDegLira**	.	.	.	4.4 [2.7; 7.0]	9.1 [2.7; 30.8]
1.8 [0.4; 7.9]	1.5 [0.4; 5.1]	1.2 [0.3; 4.2]	**HR17031**	.	.	4.6 [1.1; 18.9]	.
1.7 [0.6; 4.6]	1.4 [0.7; 2.7]	1.1 [0.6; 2.1]	0.9 [0.3; 3.5]	**iGlarLixi**	2.7 [1.0; 7.1]	2.9 [1.7; 4.8]	.
4.7 [1.2; 18.4]	3.9 [1.2; 12.2]	3.0 [0.9; 9.5]	2.6 [0.5; 13.0]	2.7 [1.0; 7.1]	**Biphasic insulin**	.	.
6.1 [2.6; 14.5]	5.0 [3.1; 8.2]	3.9 [2.5; 6.1]	3.4 [1.0; 11.4]	3.5 [2.2; 5.8]	1.3 [0.4; 3.8]	**Basal insulin**	.
15.0 [5.3; 42.6]	12.3 [4.2; 36.0]	9.6 [3.5; 25.9]	8.2 [1.7; 40.0]	8.7 [2.8; 27.1]	3.2 [0.7; 14.1]	2.5 [0.9; 6.9]	**BB insulin**

Supplementary Table 10. Exploratory univariable random-effects network meta-regression for co-primary outcomes

**Outcome**	**Moderator**	**Term**	**β (MD scale)**	**95% CI**	**p**	**Residual τ^2 (after)**	**τ^2 explained**
Change in HbA1c (%)	Baseline HbA1c	Per 1 percentage-point higher baseline HbA1c	0.8376	[0.2910, 1.3843]	0.0027	0.0434	28%
Change in HbA1c (%)	Trial duration	Per 1 week longer	0.0091	[-0.0050, 0.0232]	0.2076	0.05901	3%
Change in body weight (kg)	Baseline body weight	Per 1 kg higher baseline body weight	0.0558	[-0.0014, 0.1130]	0.0559	1.169	10%
Change in body weight (kg)	Trial duration	Per 1 week longer	0.0114	[-0.0189, 0.0417]	0.4622	1.326	0%

Abbreviations: CI, confidence interval; HbA1c, glycated hemoglobin; MD, mean difference; NMA, network meta-analysis.

Each moderator was examined separately in a univariable random-effects network meta-regression using netmetareg() from the R netmeta package. The regression coefficient β represents the change in relative MD associated with the stated increment in the study-level moderator. A positive β value indicates a more positive relative MD with increasing moderator values; interpretation of direction depends on the coding of treatment contrasts. The between-study variance in the primary NMA was $\tau^2 = 0.061$ for HbA1c and $\tau^2 = 1.309$ for body weight. The percentage of between-study variance explained was calculated as the proportional reduction in τ^2 between the primary NMA and the corresponding network meta-regression model. These analyses were post hoc, based on aggregate study-level covariates, and should be regarded as exploratory; they do not establish patient-level effect modification.

Supplementary Figure 12. Post hoc sensitivity analysis excluding the high-risk-of-bias study (DUAL V) for change in HbA1c. (A) Network plot of treatment comparisons after exclusion of DUAL V. Node size is proportional to the number of participants receiving the respective intervention, and line thickness is proportional to the number of studies contributing direct evidence; numbers on lines indicate the number of studies. **(B)** Random-effects network meta-analysis forest plot showing mean differences (MDs) with 95% confidence intervals (CIs) for change in HbA1c, with basal insulin as the reference treatment. Negative MDs favor greater HbA1c reduction. **(C)** P-scores for change in HbA1c. **(D)** Node-splitting analysis comparing direct and indirect evidence using the back-calculation method.

A

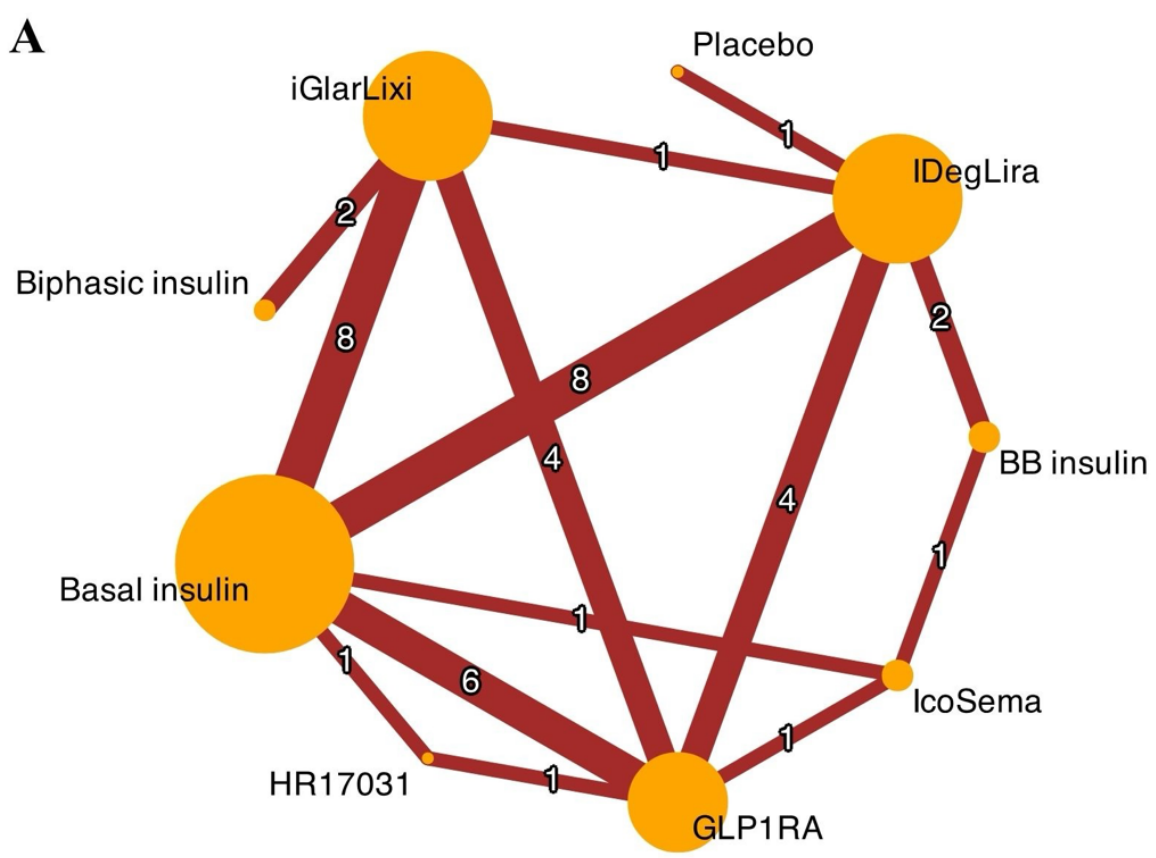

C

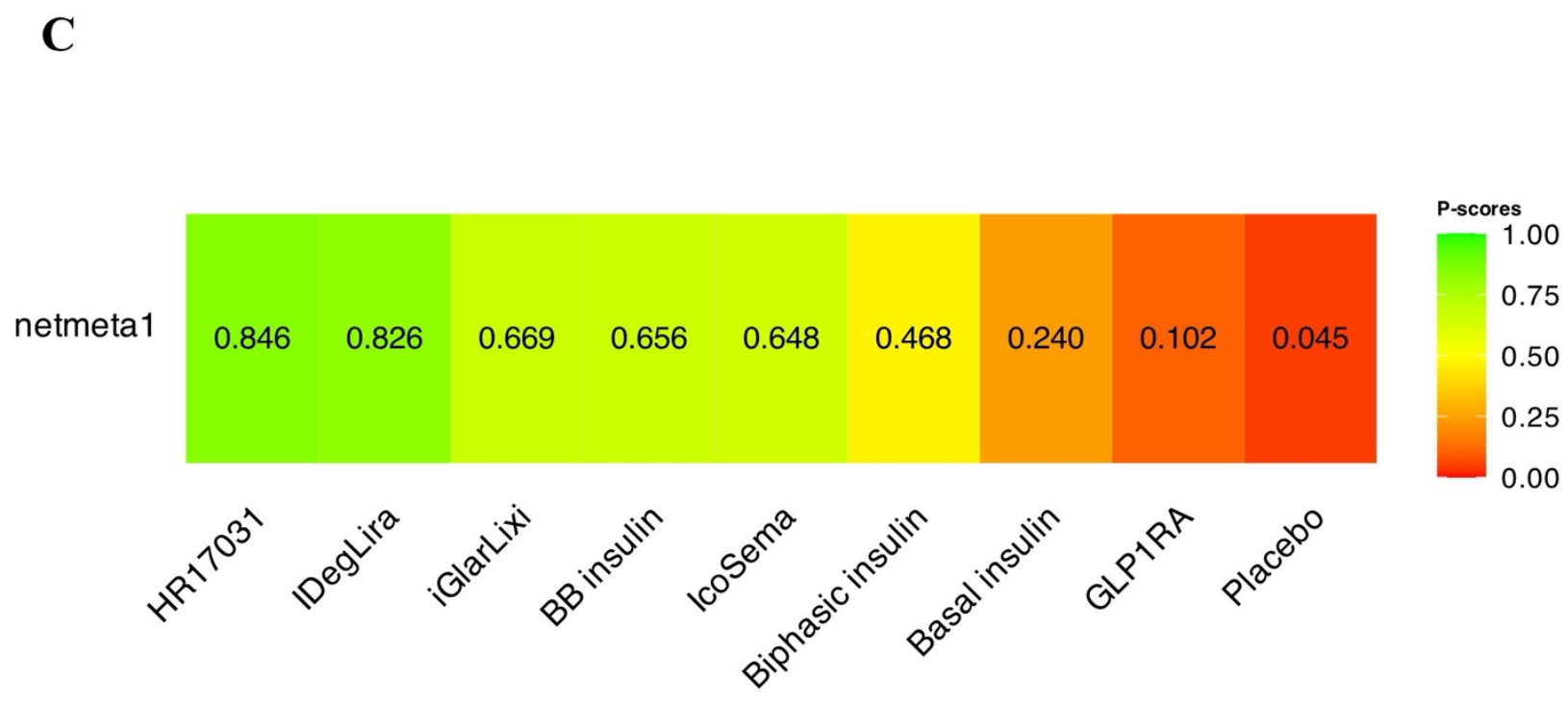

B

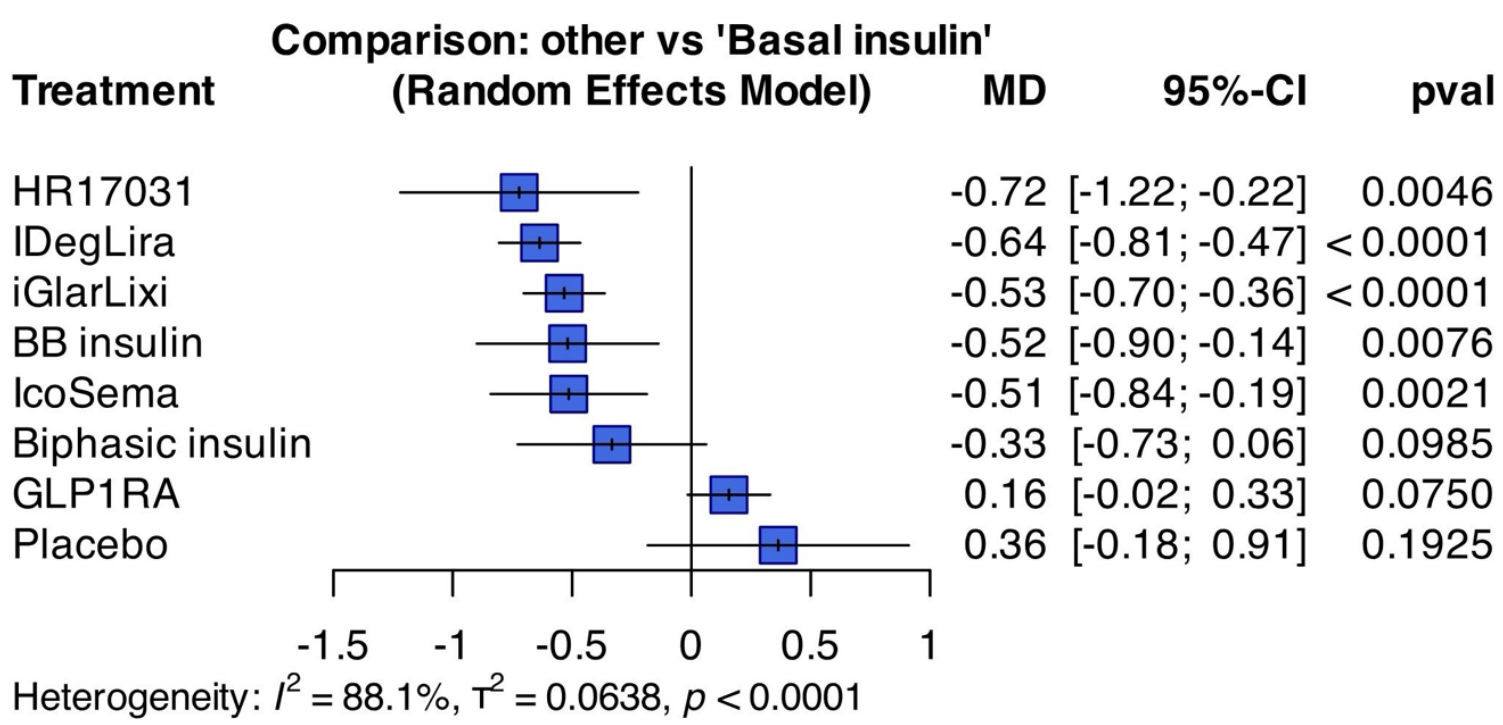

D

```
Separate indirect from direct evidence (SIDE) using back-calculation method

Random effects model:

           comparison k prop     nma  direct   indir.    Diff     z p-value
 GLP1RA:Basal insulin 6 0.65  0.1573  0.1610  0.1503  0.0107  0.06  0.9539
HR17031:Basal insulin 1 0.77 -0.7217 -0.9000 -0.1112 -0.7888 -1.30  0.1948
IcoSema:Basal insulin 1 0.43 -0.5138 -0.6600 -0.4056 -0.2544 -0.75  0.4516
IDegLira:Basal insulin 8 0.82 -0.6361 -0.6561 -0.5446 -0.1116 -0.49  0.6218
iGlarLixi:Basal insulin 8 0.81 -0.5331 -0.4570 -0.8533  0.3963  1.80  0.0720
   BB insulin:IcoSema 1 0.56 -0.0048  0.0700 -0.1015  0.1715  0.43  0.6684
  BB insulin:IDegLira 2 0.69  0.1175  0.0642  0.2356 -0.1715 -0.43  0.6684
       GLP1RA:HR17031 1 0.77  0.8790  0.7000  1.4873 -0.7873 -1.30  0.1948
       GLP1RA:IcoSema 1 0.43  0.6711  0.4500  0.8354 -0.3854 -1.13  0.2595
      GLP1RA:IDegLira 4 0.59  0.7934  0.7081  0.9156 -0.2075 -1.00  0.3153
     GLP1RA:iGlarLixi 4 0.59  0.6904  0.8377  0.4821  0.3556  1.73  0.0834
   IDegLira:iGlarLixi 1 0.06 -0.1030 -0.1000 -0.1032  0.0032  0.01  0.9948
```

Supplementary Figure 13. Post hoc sensitivity analysis excluding the high-risk-of-bias study (DUAL V) for change in body weight. (A) Network plot of treatment comparisons after exclusion of DUAL V. Node size is proportional to the number of participants receiving the respective intervention, and line thickness is proportional to the number of studies contributing direct evidence; numbers on lines indicate the number of studies. **(B)** Random-effects network meta-analysis forest plot showing MDs with 95% CIs for change in body weight, with basal insulin as the reference treatment. Negative MDs favor greater body-weight reduction. **(C)** P-scores for change in body weight. **(D)** Node-splitting analysis comparing direct and indirect evidence using the back-calculation method.

A

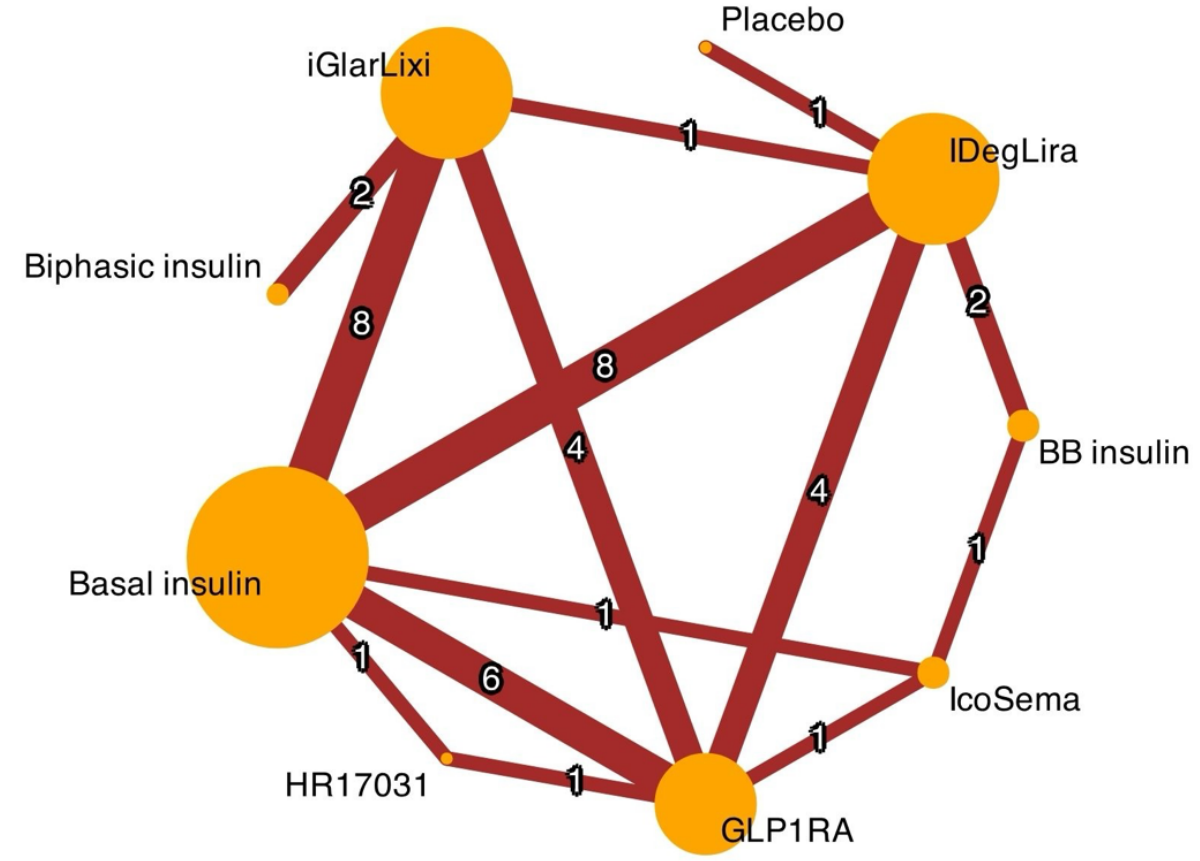

C

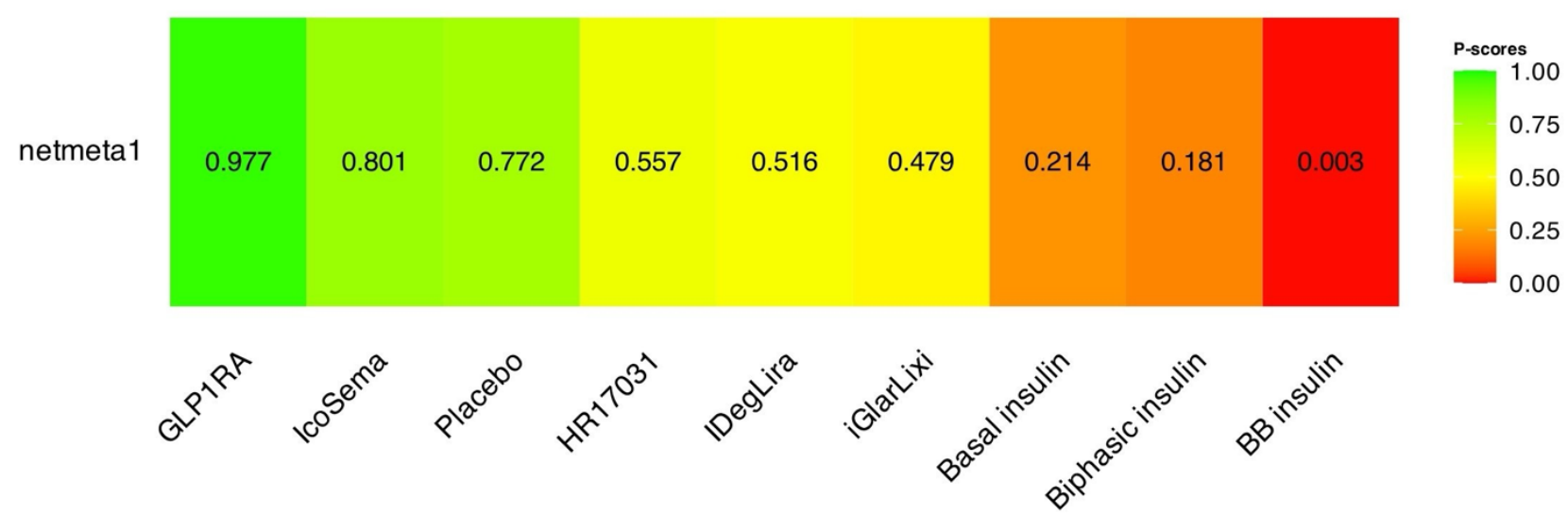

B

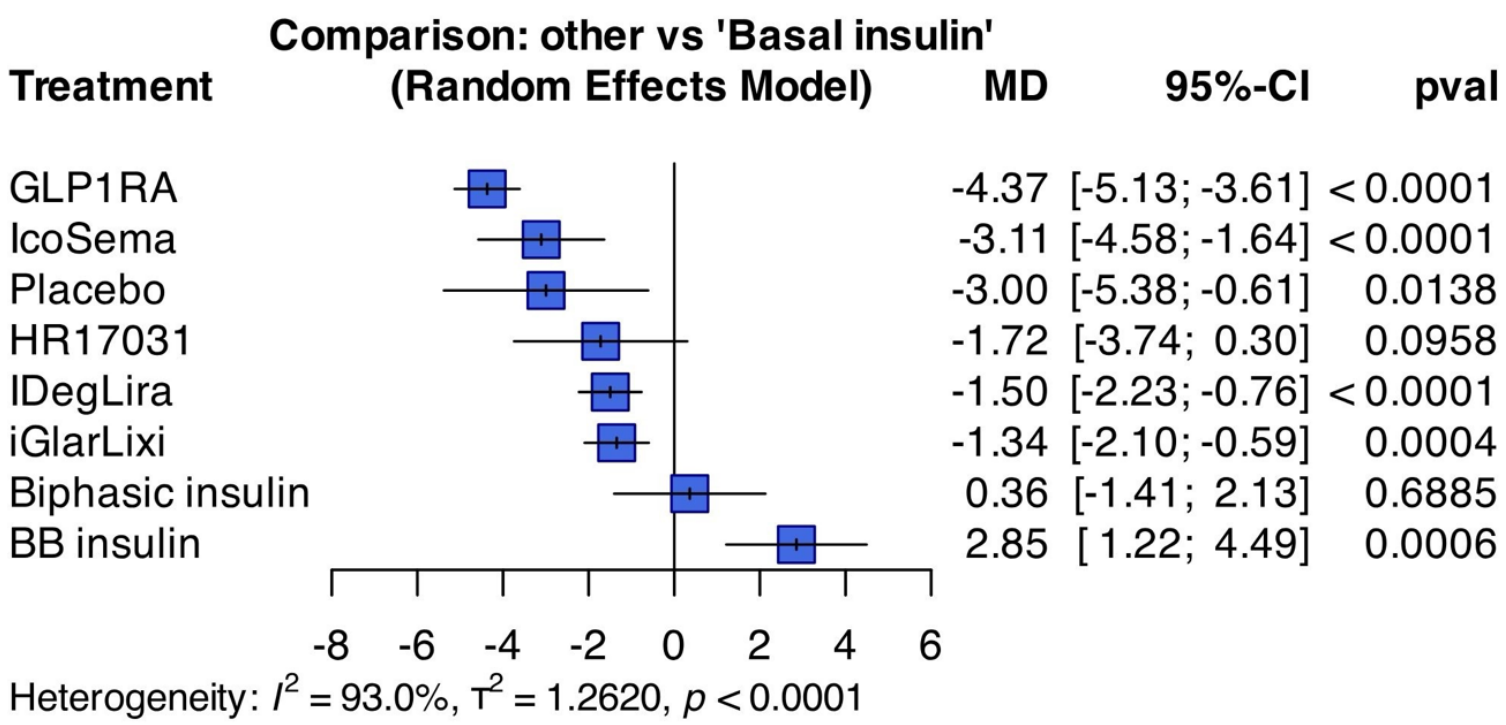

D

Separate indirect from direct evidence (SIDE) using back-calculation method

Random effects model:

comparison	k	prop	nma	direct	indir.	Diff	z	p-value
GLP1RA:Basal insulin	6	0.65	-4.3704	-4.1130	-4.8552	0.7421	0.91	0.3621
HR17031:Basal insulin	1	0.79	-1.7193	-2.1000	-0.2512	-1.8488	-0.72	0.4689
IcoSema:Basal insulin	1	0.42	-3.1071	-5.5900	-1.3128	-4.2772	-2.81	0.0049
IDegLira:Basal insulin	8	0.81	-1.4953	-1.6593	-0.8014	-0.8580	-0.90	0.3658
iGlarLixi:Basal insulin	8	0.78	-1.3450	-0.9073	-2.9014	1.9941	2.16	0.0311
BB insulin:IcoSema	1	0.54	5.9599	6.7200	5.0783	1.6417	0.93	0.3533
BB insulin:IDegLira	2	0.72	4.3481	3.8941	5.5359	-1.6417	-0.93	0.3533
GLP1RA:HR17031	1	0.74	-2.6511	-3.1000	-1.3918	-1.7082	-0.72	0.4689
GLP1RA:IcoSema	1	0.42	-1.2633	-4.5400	1.0924	-5.6324	-3.66	0.0003
GLP1RA:IDegLira	4	0.58	-2.8751	-2.9214	-2.8108	-0.1106	-0.12	0.9014
GLP1RA:iGlarLixi	4	0.58	-3.0254	-2.1766	-4.1805	2.0039	2.23	0.0256
IDegLira:iGlarLixi	1	0.10	-0.1503	0.7000	-0.2490	0.9490	0.59	0.5544
