# Supplementary material for: Glycemic control and weight loss benefits of fixed-ratio combinations of basal insulins and GLP1 receptor agonists in type 2 diabetes: A systematic review and network meta-analysis
Source: Endocrine. 2026 Oct 31;91(1):366. doi: 10.1007/s12020-026-04857-4 (PMC13723511; doi:10.1007/s12020-026-04857-4)
Supplement: Supplementary file 2 [file 12020_2026_4857_MOESM2_ESM.pdf]

Change in HbA1c

Download Report Reset

Comparison	Number of Studies	Within-study bias	Reporting bias	Indirectness	Imprecision	Heterogeneity	Incoherence	Confidence rating	Reason(s) for downgrading
**Mixed evidence**									
BB insulin vs IDegLira	2	Some concerns	Low risk	No concerns	Major concerns	No concerns	No concerns	Low	| Within-study bias | Imprecision |
BB insulin vs IcoSema	1	Some concerns	Low risk	No concerns	Major concerns	No concerns	No concerns	Low	| Within-study bias | Imprecision |
Basal insulin vs GLP1RA	6	Some concerns	Low risk	No concerns	No concerns	Major concerns	No concerns	Low	| Within-study bias | Heterogeneity |
Basal insulin vs HR17031	1	Some concerns	Low risk	No concerns	No concerns	No concerns	No concerns	Moderate	| Within-study bias |
Basal insulin vs IDegLira	9	Some concerns	Low risk	No concerns	No concerns	No concerns	No concerns	Moderate	| Within-study bias |
Basal insulin vs IcoSema	1	No concerns	Low risk	No concerns	No concerns	Major concerns	No concerns	Low	| Heterogeneity |
Basal insulin vs iGlarLixi	8	Some concerns	Low risk	No concerns	No concerns	No concerns	No concerns	Moderate	| Within-study bias |
Biphasic insulin vs iGlarLixi	2	Some concerns	Low risk	No concerns	Major concerns	No concerns	No concerns	Low	| Within-study bias | Imprecision |
GLP1RA vs HR17031	1	Some concerns	Low risk	No concerns	No concerns	No concerns	No concerns	Moderate	| Within-study bias |
GLP1RA vs IDegLira	4	Some concerns	Low risk	No concerns	No concerns	No concerns	No concerns	Moderate	| Within-study bias |
GLP1RA vs IcoSema	1	No concerns	Low risk	No concerns	No concerns	No concerns	No concerns	High	|
GLP1RA vs iGlarLixi	4	Some concerns	Low risk	No concerns	No concerns	No concerns	No concerns	Moderate	| Within-study bias |
IDegLira vs Placebo	1	Some concerns	Low risk	No concerns	No concerns	No concerns	No concerns	Moderate	| Within-study bias |
IDegLira vs iGlarLixi	1	Some concerns	Low risk	No concerns	Major concerns	No concerns	No concerns	Low	| Within-study bias | Imprecision |
**Indirect evidence**									
BB insulin vs Basal insulin	--	Some concerns	Low risk	No concerns	No concerns	Major concerns	No concerns	Very low	| Within-study bias | Heterogeneity |
BB insulin vs Biphasic insulin	--	Some concerns	Low risk	No concerns	Major concerns	No concerns	No concerns	Very low	| Within-study bias | Imprecision |
BB insulin vs GLP1RA	--	Some concerns	Low risk	No concerns	No concerns	No concerns	No concerns	Low	| Within-study bias |
BB insulin vs HR17031	--	Some concerns	Low risk	No concerns	Major concerns	No concerns	No concerns	Very low	| Within-study bias | Imprecision |
BB insulin vs Placebo	--	Some concerns	Low risk	No concerns	No concerns	No concerns	No concerns	Low	| Within-study bias |
BB insulin vs iGlarLixi	--	Some concerns	Low risk	No concerns	Major concerns	No concerns	No concerns	Very low	| Within-study bias | Imprecision |
Basal insulin vs Biphasic insulin	--	Some concerns	Low risk	No concerns	Major concerns	No concerns	No concerns	Very low	| Within-study bias | Imprecision |
Basal insulin vs Placebo	--	Some concerns	Low risk	No concerns	Major concerns	No concerns	No concerns	Very low	| Within-study bias | Imprecision |
Biphasic insulin vs GLP1RA	--	Some concerns	Low risk	No concerns	No concerns	Major concerns	No concerns	Very low	| Within-study bias | Heterogeneity |
Biphasic insulin vs HR17031	--	Some concerns	Low risk	No concerns	Major concerns	No concerns	No concerns	Very low	| Within-study bias | Imprecision |
Biphasic insulin vs IDegLira	--	Some concerns	Low risk	No concerns	Major concerns	No concerns	No concerns	Very low	| Within-study bias | Imprecision |
Biphasic insulin vs IcoSema	--	Some concerns	Low risk	No concerns	Major concerns	No concerns	No concerns	Very low	| Within-study bias | Imprecision |
Biphasic insulin vs Placebo	--	Some concerns	Low risk	No concerns	No concerns	Major concerns	No concerns	Very low	| Within-study bias | Heterogeneity |
GLP1RA vs Placebo	--	Some concerns	Low risk	No concerns	Major concerns	No concerns	No concerns	Very low	| Within-study bias | Imprecision |
HR17031 vs IDegLira	--	Some concerns	Low risk	No concerns	Major concerns	No concerns	No concerns	Very low	| Within-study bias | Imprecision |
HR17031 vs IcoSema	--	Some concerns	Low risk	No concerns	Major concerns	No concerns	No concerns	Very low	| Within-study bias | Imprecision |
HR17031 vs Placebo	--	Some concerns	Low risk	No concerns	No concerns	No concerns	No concerns	Low	| Within-study bias |
HR17031 vs iGlarLixi	--	Some concerns	Low risk	No concerns	Major concerns	No concerns	No concerns	Very low	| Within-study bias | Imprecision |
IDegLira vs IcoSema	--	Some concerns	Low risk	No concerns	Major concerns	No concerns	No concerns	Very low	| Within-study bias | Imprecision |
IcoSema vs Placebo	--	Some concerns	Low risk	No concerns	No concerns	No concerns	No concerns	Low	| Within-study bias |
IcoSema vs iGlarLixi	--	Some concerns	Low risk	No concerns	Major concerns	No concerns	No concerns	Very low	| Within-study bias | Imprecision |
Placebo vs iGlarLixi	--	Some concerns	Low risk	No concerns	No concerns	No concerns	No concerns	Low	| Within-study bias |

CINeMA is distributed, in the hope that it will be useful but without any warranty, under the GPL license. By using CINeMA you accept the following DISCLAIMER
