# Supplementary material for: Glycemic control and weight loss benefits of fixed-ratio combinations of basal insulins and GLP1 receptor agonists in type 2 diabetes: A systematic review and network meta-analysis
Source: Endocrine. 2026 Oct 31;91(1):366. doi: 10.1007/s12020-026-04857-4 (PMC13723511; doi:10.1007/s12020-026-04857-4)
Supplement: Supplementary file 3 [file 12020_2026_4857_MOESM3_ESM.pdf]

Change in Body Weight

Download Report Reset

<table>
<tr><th>Comparison</th><th>Number of Studies</th><th>Within-study bias</th><th>Reporting bias</th><th>Indirectness</th><th>Imprecision</th><th>Heterogeneity</th><th>Incoherence</th><th>Confidence rating</th><th>Reason(s) for downgrading</th></tr>
<tr><td colspan="10">Mixed evidence</td></tr>
<tr><td>BB insulin vs IDegLira</td><td>2</td><td>Some concerns</td><td>Low risk</td><td>No concerns</td><td>No concerns</td><td>No concerns</td><td>No concerns</td><td>Moderate</td><td>| Within-study bias |</td></tr>
<tr><td>BB insulin vs IcoSema</td><td>1</td><td>Some concerns</td><td>Low risk</td><td>No concerns</td><td>No concerns</td><td>No concerns</td><td>No concerns</td><td>Moderate</td><td>| Within-study bias |</td></tr>
<tr><td>Basal insulin vs GLP1RA</td><td>6</td><td>Some concerns</td><td>Low risk</td><td>No concerns</td><td>No concerns</td><td>No concerns</td><td>No concerns</td><td>Moderate</td><td>| Within-study bias |</td></tr>
<tr><td>Basal insulin vs HR17031</td><td>1</td><td>Some concerns</td><td>Low risk</td><td>No concerns</td><td>Major concerns</td><td>No concerns</td><td>No concerns</td><td>Low</td><td>| Within-study bias | Imprecision |</td></tr>
<tr><td>Basal insulin vs IDegLira</td><td>9</td><td>Some concerns</td><td>Low risk</td><td>No concerns</td><td>No concerns</td><td>Major concerns</td><td>No concerns</td><td>Low</td><td>| Within-study bias | Heterogeneity |</td></tr>
<tr><td>Basal insulin vs IcoSema</td><td>1</td><td>No concerns</td><td>Low risk</td><td>No concerns</td><td>No concerns</td><td>No concerns</td><td>Major concerns</td><td>Low</td><td>| Incoherence |</td></tr>
<tr><td>Basal insulin vs iGlarLixi</td><td>8</td><td>Some concerns</td><td>Low risk</td><td>No concerns</td><td>No concerns</td><td>Major concerns</td><td>No concerns</td><td>Low</td><td>| Within-study bias | Heterogeneity |</td></tr>
<tr><td>Biphasic insulin vs iGlarLixi</td><td>2</td><td>Some concerns</td><td>Low risk</td><td>No concerns</td><td>No concerns</td><td>Major concerns</td><td>Major concerns</td><td>Low</td><td>| Within-study bias | Heterogeneity | Incoherence |</td></tr>
<tr><td>GLP1RA vs HR17031</td><td>1</td><td>Some concerns</td><td>Low risk</td><td>No concerns</td><td>No concerns</td><td>Major concerns</td><td>No concerns</td><td>Low</td><td>| Within-study bias | Heterogeneity |</td></tr>
<tr><td>GLP1RA vs IDegLira</td><td>4</td><td>Some concerns</td><td>Low risk</td><td>No concerns</td><td>No concerns</td><td>No concerns</td><td>No concerns</td><td>Moderate</td><td>| Within-study bias |</td></tr>
<tr><td>GLP1RA vs IcoSema</td><td>1</td><td>No concerns</td><td>Low risk</td><td>No concerns</td><td>Major concerns</td><td>No concerns</td><td>Major concerns</td><td>Low</td><td>| Imprecision | Incoherence |</td></tr>
<tr><td>GLP1RA vs iGlarLixi</td><td>4</td><td>Some concerns</td><td>Low risk</td><td>No concerns</td><td>No concerns</td><td>No concerns</td><td>No concerns</td><td>Moderate</td><td>| Within-study bias |</td></tr>
<tr><td>IDegLira vs Placebo</td><td>1</td><td>Some concerns</td><td>Low risk</td><td>No concerns</td><td>Major concerns</td><td>No concerns</td><td>Major concerns</td><td>Low</td><td>| Within-study bias | Imprecision | Incoherence |</td></tr>
<tr><td>IDegLira vs iGlarLixi</td><td>1</td><td>Some concerns</td><td>Low risk</td><td>No concerns</td><td>Major concerns</td><td>No concerns</td><td>No concerns</td><td>Low</td><td>| Within-study bias | Imprecision |</td></tr>
<tr><td colspan="10">Indirect evidence</td></tr>
<tr><td>BB insulin vs Basal insulin</td><td>--</td><td>Some concerns</td><td>Low risk</td><td>No concerns</td><td>No concerns</td><td>Major concerns</td><td>Major concerns</td><td>Low</td><td>| Within-study bias | Heterogeneity | Incoherence |</td></tr>
<tr><td>BB insulin vs Biphasic insulin</td><td>--</td><td>Some concerns</td><td>Low risk</td><td>No concerns</td><td>Major concerns</td><td>No concerns</td><td>Major concerns</td><td>Very low</td><td>| Within-study bias | Imprecision | Incoherence |</td></tr>
<tr><td>BB insulin vs GLP1RA</td><td>--</td><td>Some concerns</td><td>Low risk</td><td>No concerns</td><td>No concerns</td><td>No concerns</td><td>Major concerns</td><td>Low</td><td>| Within-study bias | Incoherence |</td></tr>
<tr><td>BB insulin vs HR17031</td><td>--</td><td>Some concerns</td><td>Low risk</td><td>No concerns</td><td>No concerns</td><td>No concerns</td><td>Major concerns</td><td>Low</td><td>| Within-study bias | Incoherence |</td></tr>
<tr><td>BB insulin vs Placebo</td><td>--</td><td>Some concerns</td><td>Low risk</td><td>No concerns</td><td>No concerns</td><td>No concerns</td><td>Major concerns</td><td>Low</td><td>| Within-study bias | Incoherence |</td></tr>
<tr><td>BB insulin vs iGlarLixi</td><td>--</td><td>Some concerns</td><td>Low risk</td><td>No concerns</td><td>No concerns</td><td>No concerns</td><td>Major concerns</td><td>Low</td><td>| Within-study bias | Incoherence |</td></tr>
<tr><td>Basal insulin vs Biphasic insulin</td><td>--</td><td>Some concerns</td><td>Low risk</td><td>No concerns</td><td>Major concerns</td><td>No concerns</td><td>Major concerns</td><td>Very low</td><td>| Within-study bias | Imprecision | Incoherence |</td></tr>
<tr><td>Basal insulin vs Placebo</td><td>--</td><td>Some concerns</td><td>Low risk</td><td>No concerns</td><td>No concerns</td><td>Major concerns</td><td>Major concerns</td><td>Low</td><td>| Within-study bias | Heterogeneity | Incoherence |</td></tr>
<tr><td>Biphasic insulin vs GLP1RA</td><td>--</td><td>Some concerns</td><td>Low risk</td><td>No concerns</td><td>No concerns</td><td>No concerns</td><td>Major concerns</td><td>Low</td><td>| Within-study bias | Incoherence |</td></tr>
<tr><td>Biphasic insulin vs HR17031</td><td>--</td><td>Some concerns</td><td>Low risk</td><td>No concerns</td><td>Major concerns</td><td>No concerns</td><td>Major concerns</td><td>Very low</td><td>| Within-study bias | Imprecision | Incoherence |</td></tr>
<tr><td>Biphasic insulin vs IDegLira</td><td>--</td><td>Some concerns</td><td>Low risk</td><td>No concerns</td><td>No concerns</td><td>Major concerns</td><td>Major concerns</td><td>Low</td><td>| Within-study bias | Heterogeneity | Incoherence |</td></tr>
<tr><td>Biphasic insulin vs IcoSema</td><td>--</td><td>Some concerns</td><td>Low risk</td><td>No concerns</td><td>No concerns</td><td>No concerns</td><td>Major concerns</td><td>Low</td><td>| Within-study bias | Incoherence |</td></tr>
<tr><td>Biphasic insulin vs Placebo</td><td>--</td><td>Some concerns</td><td>Low risk</td><td>No concerns</td><td>No concerns</td><td>Major concerns</td><td>Major concerns</td><td>Low</td><td>| Within-study bias | Heterogeneity | Incoherence |</td></tr>
<tr><td>GLP1RA vs Placebo</td><td>--</td><td>Some concerns</td><td>Low risk</td><td>No concerns</td><td>Major concerns</td><td>No concerns</td><td>Major concerns</td><td>Very low</td><td>| Within-study bias | Imprecision | Incoherence |</td></tr>
<tr><td>HR17031 vs IDegLira</td><td>--</td><td>Some concerns</td><td>Low risk</td><td>No concerns</td><td>Major concerns</td><td>No concerns</td><td>Major concerns</td><td>Very low</td><td>| Within-study bias | Imprecision | Incoherence |</td></tr>
<tr><td>HR17031 vs IcoSema</td><td>--</td><td>Some concerns</td><td>Low risk</td><td>No concerns</td><td>Major concerns</td><td>No concerns</td><td>Major concerns</td><td>Very low</td><td>| Within-study bias | Imprecision | Incoherence |</td></tr>
<tr><td>HR17031 vs Placebo</td><td>--</td><td>Some concerns</td><td>Low risk</td><td>No concerns</td><td>Major concerns</td><td>No concerns</td><td>Major concerns</td><td>Very low</td><td>| Within-study bias | Imprecision | Incoherence |</td></tr>
<tr><td>HR17031 vs iGlarLixi</td><td>--</td><td>Some concerns</td><td>Low risk</td><td>No concerns</td><td>Major concerns</td><td>No concerns</td><td>Major concerns</td><td>Very low</td><td>| Within-study bias | Imprecision | Incoherence |</td></tr>
<tr><td>IDegLira vs IcoSema</td><td>--</td><td>Some concerns</td><td>Low risk</td><td>No concerns</td><td>Major concerns</td><td>No concerns</td><td>Major concerns</td><td>Very low</td><td>| Within-study bias | Imprecision | Incoherence |</td></tr>
<tr><td>IcoSema vs Placebo</td><td>--</td><td>Some concerns</td><td>Low risk</td><td>No concerns</td><td>Major concerns</td><td>No concerns</td><td>Major concerns</td><td>Very low</td><td>| Within-study bias | Imprecision | Incoherence |</td></tr>
<tr><td>IcoSema vs iGlarLixi</td><td>--</td><td>Some concerns</td><td>Low risk</td><td>No concerns</td><td>No concerns</td><td>Major concerns</td><td>Major concerns</td><td>Low</td><td>| Within-study bias | Heterogeneity | Incoherence |</td></tr>
<tr><td>Placebo vs iGlarLixi</td><td>--</td><td>Some concerns</td><td>Low risk</td><td>No concerns</td><td>Major concerns</td><td>No concerns</td><td>Major concerns</td><td>Very low</td><td>| Within-study bias | Imprecision | Incoherence |</td></tr>
</table>

CINeMA is distributed, in the hope that it will be useful but without any warranty, under the AGPLv3 license. By using CINeMA you accept the following DISCLAIMER
